# Generation of HepG2 Cells with High Expression of Multiple Drug-Metabolizing Enzymes for Drug Discovery Research Using a PITCh System

**DOI:** 10.3390/cells11101677

**Published:** 2022-05-18

**Authors:** Ryosuke Negoro, Mitsuki Tasaka, Sayaka Deguchi, Kazuo Takayama, Takuya Fujita

**Affiliations:** 1 Laboratory of Molecular Pharmacokinetics, College of Pharmaceutical Sciences, Ritsumeikan University, 1-1-1 Noji-Higashi, Kusatsu 525-8577, Japan; fujita-t@ph.ritsumei.ac.jp; 2 Laboratory of Molecular Pharmacokinetics, Graduate School of Pharmaceutical Sciences, Ritsumeikan University, 1-1-1 Noji-Higashi, Kusatsu 525-8577, Japan; ph0124rh@ed.ritsumei.ac.jp; 3 Center for iPS Cell Research and Application (CiRA), Kyoto University, Kyoto 606-8507, Japan; sayaka.deguchi@cira.kyoto-u.ac.jp (S.D.); kazuo.takayama@cira.kyoto-u.ac.jp (K.T.); 4 Japan Agency for Medical Research and Development-Core Research for Evolutionary Medical Science and Technology (AMED-CREST), Japan Agency for Medical Research and Development, 1-7-1 Otemachi, Chiyoda-ku, Tokyo 100-0004, Japan; 5 Research Center for Drug Discovery and Development, Ritsumeikan University, 1-1-1 Noji-Higashi, Kusatsu 525-8577, Japan

**Keywords:** genome editing, CRISPR-Cas9, PITCh system, HepG2 cell, primary human hepatocytes, CYP1A2, CYP2C9, CYP2C19, CYP2D6, CYP3A4, POR, UGT1A1, drug-metabolizing enzyme

## Abstract

HepG2 cells are an inexpensive hepatocyte model that can be used for repeated experiments, but HepG2 cells do not express major cytochrome P450s (CYPs) and UDP glucuronosyltransferase family 1 member A1 (UGT1A1). In this study, we established CYP3A4–POR–UGT1A1–CYP1A2–CYP2C19–CYP2C9–CYP2D6 (CYPs–UGT1A1) knock-in (KI)-HepG2 cells using a PITCh system to evaluate whether they could be a new hepatocyte model for pharmaceutical studies. To evaluate whether CYPs–UGT1A1 KI-HepG2 cells express and function with CYPs and UGT1A1, gene expression levels of *CYPs* and *UGT1A1* were analyzed by using real-time PCR, and metabolites of CYPs or UGT1A1 substrates were quantified by HPLC. The expression levels of *CYPs* and *UGT1A1* in the CYPs–UGT1A1 KI-HepG2 cells were comparable to those in primary human hepatocytes (PHHs) cultured for 48 h. The CYPs and UGT1A1 activity levels in the CYPs–UGT1A1 KI-HepG2 cells were much higher than those in the wild-type (WT)-HepG2 cells. These results suggest that the CYPs–UGT1A1 KI-HepG2 cells expressed functional CYPs and UGT1A1. We also confirmed that the CYPs–UGT1A1 KI-HepG2 cells were more sensitive to drug-induced liver toxicity than the WT-HepG2 cells. CYPs–UGT1A1 KI-HepG2 cells could be used to predict drug metabolism and drug-induced liver toxicity, and they promise to be a helpful new hepatocyte model for drug discovery research.

## 1. Introduction

An in vitro hepatocyte model for evaluation of drug metabolism and drug-induced liver injury would be useful for drug discovery research [1,2,3]. Most drugs used in clinical practice are metabolized by one of five cytochrome P450s (CYPs): cytochrome P450 family 1 subfamily A member 2 (CYP1A2), cytochrome P450 family 2 subfamily C member 9 (CYP2C9), cytochrome P450 family 2 subfamily C member 19 (CYP2C19), cytochrome P450 family 2 subfamily D member 6 (CYP2D6) and cytochrome P450 family 3 subfamily A member 4 (CYP3A4) [4,5], all associated with phase I, or drug-activating metabolic reactions. The role of P450 oxidoreductase (POR, a coenzyme of CYPs) is also important in CYPs-mediated drug metabolism [6,7]. Drugs metabolized by cytochrome P450 ultimately undergo conjugation reactions by conjugation enzymes such as UDP glucuronosyltransferase family 1 member A1 (UGT1A1); these are phase II, or drug-inactivating metabolic reactions [5]. Therefore, hepatocyte models that can predict effects of both types of critical drug-metabolizing enzymes are desirable. Currently, primary human hepatocytes (PHHs) are the gold standard for in vitro hepatocyte models [8,9]. Drug metabolism and drug-induced liver injury can be accurately predicted using PHHs [8]. However, PHHs have problems such as lot-to-lot variations, high cost, and difficulty to passage. Furthermore, PHHs cannot be cultured for a long time because their drug-metabolizing activity is weakened immediately after the start of culture [1,10,11]. Recently, culture methods for human liver organoids have been established, enabling passage and long-term culture [12,13,14]. However, human liver organoids are problematic due to their high culture cost and difficulty with mass culture. On the other hand, human hepatoblastoma cell line-derived HepG2 cells are an inexpensive in vitro hepatocyte model that can be used for repeated experiments, but HepG2 cells do not express major CYPs or UGT1A1 [15,16,17]. Thus, HepG2 cells can not accurately predict drug metabolism and the potential for drug-induced liver injury.

Recently, human embryonic stem/induced pluripotent stem (ES/iPS) cell-derived hepatocyte-like cells have attracted attention as a new in vitro model for pharmaceutical studies investigating drug metabolism and the potential for drug-induced liver injury [18]. In addition, since human iPS cells can be established from various donors, human iPS cell-derived hepatocyte-like cells are expected to be used to predict potential differences in drug metabolism caused by SNPs [19,20]. However, human iPS cell-derived hepatocyte-like cells are immature and have low expression levels of major CYPs and UGT1A1 [21,22,23]. Moreover, human ES/iPS cell-derived hepatocyte-like cells cannot be used for large-scale drug metabolism and drug-induced liver injury experiments because of their high cost and difficulty to passage. An inexpensive and repeatable in vitro hepatocyte model for assessing drug metabolism and drug-induced liver injury has not yet been constructed.

In our prior research, we successfully expressed multiple drug-metabolizing enzymes in human colon carcinoma-derived Caco-2 cells using the Precise Integration into Target Chromosomes (PITCh) system based on the clustered regularly interspaced short palindromic repeats-CRISPRassociated 9 (CRISPR-Cas9) system [24]. Furthermore, we showed that multiple drug-metabolizing enzymes expressing Caco-2 cells can be used for pharmacokinetic studies in the small intestine [24]. In this study, we used the PITCh system to insert CYP1A2, CYP2C9, CYP2C19, CYP2D6, CYP3A4, POR, and UGT1A1 gene expression cassettes, which play critical roles in drug metabolism, into HepG2 cells to evaluate whether they can be a new in vitro human hepatocyte model in pharmaceutical studies of drug metabolism and the potential for drug-induced liver injury.

## 2. Materials and Methods

### 2.1. Materials

Sulfaphenazole, S-mephenytoin, bufuralol, 4′-hydroxy diclofenac, 4′-hydroxy mephenytoin, 1′-hydroxy bufuralol, 1′-hydroxy midazolam, 7′-hydroxy coumarin glucuronide, 7′-hydroxy coumarin sulfate, 4′-hydroxy propranolol, and 5′-hydroxy propranolol were purchased from Cayman Chemical (Ann Arbor, MI, USA). Desisopropylpropranolol was purchased from Toronto Research Chemicals (North York, ON, Canada). Puromycin, G418, hygromycin B, midazolam, propranolol, and troglitazone were purchased from FUJIFILM Wako (Tokyo, Japan). Zeocin™ was purchased from InvivoGen (San Diego, CA, USA). Terbinafine, itraconazole, phenacetin, diclofenac, 7′-hydroxy coumarin, acetaminophen, amiodarone, benzbromarone, and imipramine were purchased from Tokyo Kasei (Tokyo, Japan). Dulbecco’s Modified Eagle’s Medium (DMEM), Minimum Essential Medium Non-Essential Amino Acids Solution, Antibiotic–Antimycotic Mixed Stock Solution, Sepasol^®^-RNA I Super G, and Cell Count Reagent SF were purchased from Nacalai Tesque (Kyoto, Japan). ISOGEN was purchased from NIPPON GENE (Kyoto, Japan). PowerUp SYBR Green Master Mix was purchased from Thermo Fisher Scientific (Waltham, WA, USA). The ReveTra Ace^®^ qPCR RT kit was purchased from Toyobo (Osaka, Japan). Fetal bovine serum (FBS) was purchased from Sigma-Aldrich (Munich, Germany). Tks Gflex DNA polymerase was purchased from Takara Bio (Kusatsu, Japan). Collagen I was purchased from Corning (New York, NY, USA). PEI MAX^TM^-Transfection Grade Linear Polyethylenimine Hydrochloride (MW, 40,000) was purchased from Polysciences (Warrington, PA, USA). All other reagents were purchased as commercially available.

### 2.2. HepG2 Cells

The human hepatoblastoma cell line, HepG2 cells (RCB1648), was provided by the RIKEN BRC through the National BioResource Project of the MEXT/AMED, Japan. The HepG2 cells were cultured with DMEM containing 10% FBS, 1% Minimum Essential Medium Non-Essential Amino Acids Solution, and 1% Antibiotic–Antimycotic Mixed Stock Solution.

### 2.3. Primary Human Hepatocytes

Cryopreserved human hepatocytes (HUCPI, Lonza) were used in this study. The PHH culture protocol was described previously [25,26]. The human hepatocytes were seeded at 1.0 × 10^5^ cells/cm^2^ onto collagen I-coated 48-well plates. The PHHs cultured for 48 h after plating were used in the experiments.

### 2.4. Vector Construction

In accordance with a previously described protocol [27,28], pX330A-AAVS1/PITCh, pX330A-CCR5/PITCh, pX330A-CYP3A7/PITCh, and pX330A-hROSA26/PITCh, the all-in-one CRISPR-Cas9 vector system for cutting the genomic *AAVS1*, *CCR5, CYP3A7*, or *hROSA26* loci were constructed using the plasmids pX330A-1×2 (Addgene plasmid No. 58766: http://n2t.net/addgene:58766 (accessed on 7 March 2022); RRID: Addgene_58766, a gift from Dr. Takashi Yamamoto) [27] and pX330S-2-PITCh (Addgene plasmid No. 63670: http://n2t.net/addgene:63670; RRID (accessed on 7 March 2022): Addgene_63670, a gift from Dr. Takashi Yamamoto) [28]. The oligonucleotides for the sgRNA template targeting *AAVS1*, *CCR5*, *CYP3A7*, and *hROSA26* are listed in the Supplemental Information (Supplemental Experimental Procedures).

Donor vectors were constructed according to a protocol described previously [24]. The CYP1A2 (NM_000761.5), CYP2C9 (NM_000771.4), CYP2C19 (NM_000769.4), CYP2D6 (NM_000106.6), CYP3A4 (NM_017460.6), POR (NM_001367562.1), and UGT1A1 (NM_000463.2) genes were amplified by Tks Gflex DNA polymerase. The donor plasmid sequences are described in the Supplemental Information (Supplementary Sequences 1).

### 2.5. Generation of CYPs–UGT1A1 KI-HepG2 Cells

The HepG2 cells were seeded in 12-well plates at 1.0 × 10^5^ cells/well. The next day, 3.2 μg of the CRISPR-Cas9 plasmids and donor plasmids were co-transfected into the HepG2 cells using 8 μg PEI MAX^TM^-Transfection Grade Linear Polyethylenimine Hydrochloride (MW, 40,000). After culturing for 2 days, the medium was replaced with a 5 μg/mL puromycin-, 4 mg/mL G418-, 0.5 mg/mL Zeocin™-, or 0.4 mg/mL hygromycin B-containing medium. Several weeks after the transfection, several individual colonies were picked up.

### 2.6. Real-Time RT-PCR

Total RNA was isolated from the HepG2 cells using Sepasol^®^-RNA I Super G. Total RNA in the PHHs was isolated using ISOGEN. According to the manufacturer’s protocol, cDNA was synthesized with ReveTra Ace^®^ qPCR RT kit. The real-time RT-PCR protocol was described previously [24].

### 2.7. HPLC

The cells were cultured on 48-well plates, then washed with pH 7.4 HBSS containing 10 mM HEPES and 25 mM glucose. The cells were preincubated for 30 min with pH 7.4 HBSS containing 10 mM HEPES and 25 mM glucose in the presence or absence of the CYP2C9, CYP2D6, or CYP3A4 inhibitor, 10 μM sulfaphenazole, 10 μM terbinafine, or 10 μM itraconazole, respectively. After preincubation, HBSS containing 10 μM phenacetin, 10 μM diclofenac, 50 μM S-mephenytoin, 1 μM bufuralol, 10 μM midazolam, 10 μM 7′-hydroxy coumarin, or 1 μM propranolol was added, and the cells were incubated at 37 ℃ for 360 min. The metabolites of the substrates are acetaminophen, 4′-hydroxy diclofenac, 4′-hydroxy mephenytoin, 1′-hydroxy bufuralol, 1′-hydroxy midazolam, 7′-hydroxy coumarin glucuronide, 7′-hydroxy coumarin sulfate, 4′-hydroxy propranolol, 5′-hydroxy propranolol, and desisopropylpropranolol. The samples were collected from the supernatant, then immediately mixed with the same volume of acetonitrile. The mixed solutions were centrifuged for 5 min at 15,000× *g*. The samples were filtrated with a 0.45 μm syringe filter (Merck Millipore), then analyzed by HPLC to measure the concentration of acetaminophen, 4′-hydroxy diclofenac, 4′-hydroxy mephenytoin, 1′-hydroxy bufuralol, 1′-hydroxy midazolam, 7′-hydroxy coumarin glucuronide, 7′-hydroxy coumarin sulfate, 4′-hydroxy propranolol, 5′-hydroxy propranolol, or desisopropylpropranolol according to the standard curve. HPLC analysis was performed using a LO-20AD SPD + RF (DGU-20A, LC-20AD, RF-20A xs, SIL-20AC, CBM-20A, SPD-20A, CTO-20AC; Shimadzu). The HPLC methods are listed in Table S1. The concentrations of each metabolite were calculated according to the respective standard followed by normalization to the protein content per well.

### 2.8. Cell Viability Tests

The HepG2 cells were seeded in 96-well plates at 1.0 × 10^4^ cells/well. The next day, the HepG2 cells were treated with various concentrations of amiodarone, benzbromarone, acetaminophen, imipramine, or troglitazone. After 24 h, to examine the cell viability, we performed a WST-8 (2-(2-methoxy-4-nitrophenyl)-3-(4-nitrophenyl)-5-(2,4-disulfophenyl)-2H-tetrazolium, monosodium salt) assay by using Cell Count Reagent SF according to the manufacturer’s instructions. The cell viability was calculated as the percentage of that in the cells treated with a vehicle only.

### 2.9. Statistical Analysis

Statistical analyses were performed as indicated in figure legends using the v. 1.55 Easy R (EZR) software. A value of *p* < 0.05 was considered statistically significant. Using the SigmaPlot v. 14.5 statistical software (Systat Software, San Jose, CA, USA), 50% inhibitory concentrations (IC50) were determined by use of nonlinear regression analysis.

## 3. Results

### 3.1. Generation of CYPs–UGT1A1 KI-HepG2 Cells

**Figure 1:**
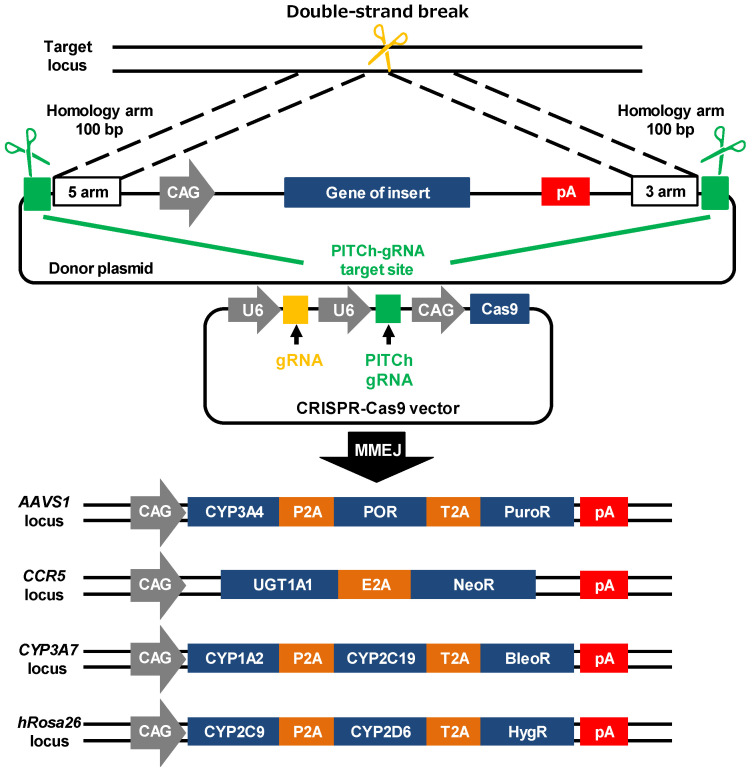
**Generation of CYPs–UGT1A1 KI-HepG2 cells.** This schematic overview shows the targeting strategy for *AAVS1*, *CCR5*, *CYP3A7*, or *hROSA26* loci. Donor and CRISPR-Cas9 plasmids: CAG, cytomegalovirus (CMV) early enhancer/chicken β actin promoter; U6, U6 promoter; PITCh, Precise Integration into Target Chromosome; MMEJ, microhomology-mediated end joining; AAVS1, adeno-associated virus integration site 1; CCR5, C–C chemokine receptor type 5; CYP3A7, cytochrome P450 family 3 subfamily A member 7; hROSA26, human ROSA26; P2A, self-cleaving P2A peptide sequence; T2A, self-cleaving T2A peptide sequence; E2A, self-cleaving E2A peptide sequence; CYP1A2, cytochrome P450 family 1 subfamily A member 2; CYP2C9, cytochrome P450 family 2 subfamily C member 9; CYP2C19; cytochrome P450 family 2 subfamily C member 19; CYP2D6, cytochrome P450 family 2 subfamily D member 6; CYP3A4, cytochrome P450 family 3 subfamily A member 4; POR, P450 oxidoreductase; UGT1A1, UDP glucuronosyltransferase family 1 member A1; PuroR, puromycin resistance protein; NeoR, neomycin resistance protein; BleoR, bleomycin resistance protein; HygR, hygromycin B resistance protein; pA, polyadenylation sequence.

In order to generate *CYP3A4–POR–UGT1A1–CYP1A2–CYP2C19–CYP2C9–CYP2D6* (CYPs–UGT1A1) knock-in (KI)-HepG2 cells, genome-editing experiments were conducted four times. The schematic overview shows the targeting strategy for the *AAVS1*, *CCR5*, *CYP3A7*, and *hROSA26* loci (Figure 1). Firstly, we inserted the CAG–*CYP3A4–POR*–*PuroR R*-pA cassette into the *AAVS1* locus of the wild-type (WT)-HepG2 cells using the PITCh system. After puromycin selection, puromycin-resistant *CYP3A4–POR* KI-HepG2 cell clones were obtained. Secondly, we inserted the CAG–*UGT1A1*–*NeoR*-pA cassette into the *CCR5* locus of the *CYP3A4–POR* KI-HepG2 cells. After G418 selection, *CYP3A4*–*POR*–UGT1A1 KI-HepG2 cell clones were obtained. Thirdly, we inserted the CAG–*CYP1A2–CYP2C19*–*BleoR*-pA cassette into the *CYP3A7* locus of the *CYP3A4–POR–UGT1A1* KI-HepG2 cells. After Zeocin™ selection, *CYP3A4–POR–UGT1A1–CYP1A2–CYP2C19* KI-HepG2 cell clones were obtained. Finally, we inserted the CAG–*CYP2C9–CYP2D6*–*HygR*-pA cassette into the *hROSA26* locus of the *CYP3A4–POR–UGT1A1–CYP1A2–CYP2C19* KI-HepG2 cells. After hygromycin B selection, CYPs–UGT1A1 KI-HepG2 cell clones were obtained.

**Figure 2:**
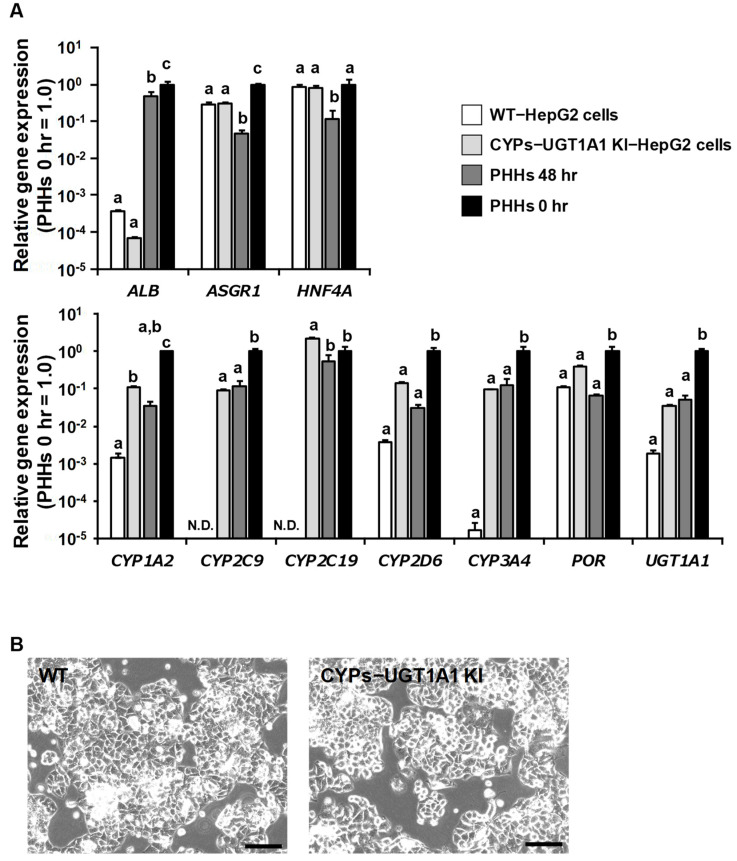
**Expression analysis of the CYPs–UGT1A1 KI-HepG2 cells.** (**A**) Real time RT-PCR was used to determine expression levels of genes for *albumin* *(ALB)*, *asialoglycoprotein receptor 1* (*ASGR1*), and *hepatocyte nuclear factor 4 alpha* (*HNF4A*), which served as hepatic markers, and for the drug-metabolizing enzymes *cytochrome P450 family 1 subfamily A member 2* (*CYP1A2*), *cytochrome P450 family 2 subfamily C member 9* (*CYP2C9*), *cytochrome P450 family 2 subfamily C member 19* (*CYP2C19*), *cytochrome P450 family 2 subfamily D member 6* (*CYP2D6*), *cytochrome P450 family 3 subfamily A member 4* (*CYP3A4*), *P450 oxidoreductase* (*POR*), and *UDP glucuronosyltransferase family 1 member A1* (*UGT1A1*) in the WT-HepG2 cells, CYPs–UGT1A1 KI-HepG2 cells, 48 h-cultured primary human hepatocytes (PHHs 48 h), and PHHs collected immediately after thawing (PHHs 0 h). On the y-axis, the gene expression levels in the PHHs 0 h were taken as 1.0. The data represent the means ± SD (*n* = 3, technical replicates). N.D., not detected. Statistical significance was evaluated by one-way ANOVA followed by Tukey’s post-hoc test (*p* < 0.05). The groups that do not share the same letter had significantly different results. (**B**) Phase-contrast images of the WT-HepG2 cells and the CYPs–UGT1A1 KI-HepG2 cells are shown. Scale bars represent 100 μm.

To evaluate whether the CYPs–UGT1A1 KI-HepG2 cells were negatively affected by the genome-editing experiments, we used real-time PCR to analyze the gene expression levels of albumin *(ALB*), asialoglycoprotein receptor 1 (*ASGR1*), and hepatocyte nuclear factor 4 alpha (*HNF4A*) as hepatic markers (Figure 2A). We used 48 h cultured primary human hepatocytes (PHHs 48 h) and PHHs collected immediately after thawing (PHHs 0 h) as positive controls. The expression levels of hepatic marker genes in the CYPs–UGT1A1 KI-HepG2 cells were similar to those in the WT-HepG2 cells. The expression levels of drug-metabolizing enzyme genes (*CYP1A2*, *CYP2C9*, *CYP2C19*, *CYP2D6*, *CYP3A4*, *POR*, and *UGT1A1*) in the CYPs–UGT1A1 KI-HepG2 cells were comparable to those in the PHHs 48 h (Figure 2A). In the CYPs–UGT1A1 KI-HepG2 cells, there was no decrease in the expression levels of drug-metabolizing enzymes during passages 38 to 57 (Figure S1).

Phase images showed that there was no morphologic difference between the WT-HepG2 cells and the CYPs–UGT1A1 KI-HepG2 cells (Figure 2B). These results suggest that the knocking-in of CYPs and UGT1A1 does not negatively affect HepG2 cells.

### 3.2. Drug-Metabolizing Activity Evaluation of the CYPs–UGT1A1 KI-HepG2 Cells

**Figure 3:**
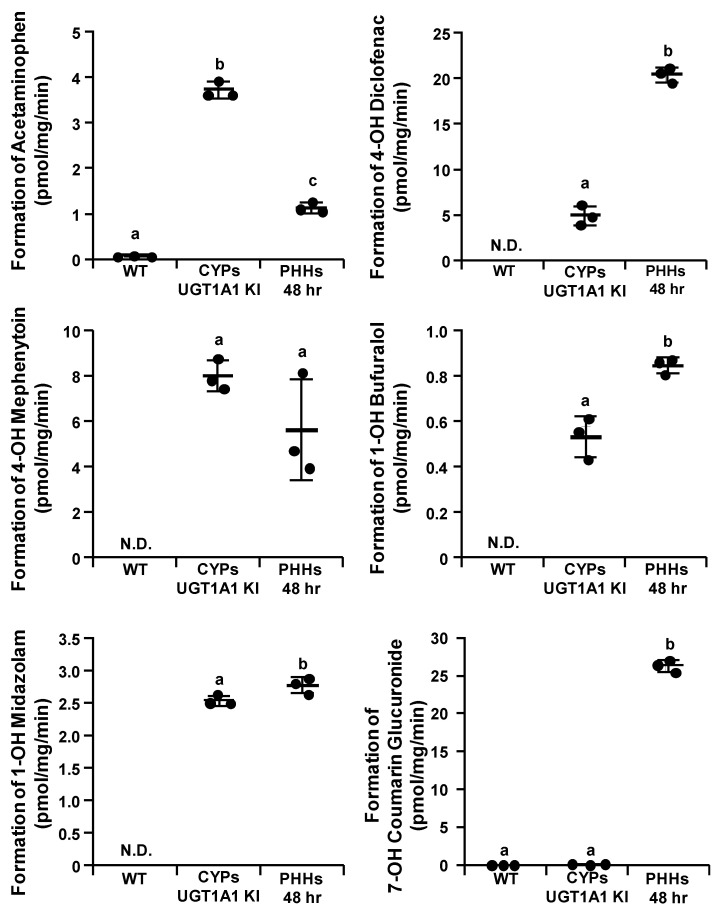
**Drug-metabolizing activity evaluation of the CYPs–UGT1A1 KI-HepG2 cells.** The CYP1A2, CYP2C9, CYP2C19, CYP2D6, CYP3A4, and UGT1A1 activities in the WT-HepG2 cells, CYPs–UGT1A1 KI-HepG2 cells, and 48 h-cultured primary human hepatocytes (PHHs 48 h) were examined by quantifying the metabolites of CYP and UGT substrates (10 μM phenacetin, 10 μM diclofenac, 50 μM S-mephenytoin, 1 μM bufuralol, 10 μM midazolam, and 10 μM 7′-hydroxy coumarin; these compounds are substrates for CYP1A2, 2C9, 2C19, 2D6, 3A4, and UGTs, respectively). The quantity of metabolites (acetaminophen, 4′-hydroxy diclofenac, 4′-hydroxy mephenytoin, 1′-hydroxy bufuralol, 1′-hydroxy midazolam and 7′-hydroxy coumarin glucuronide; these compounds are metabolites for CYP1A2, 2C9, 2C19, 2D6, 3A4 and UGTs, respectively) were measured by HPLC. Data represent the means ± SD (*n* = 3, technical replicates). N.D., not detected. Statistical significance was evaluated by one-way ANOVA followed by Tukey’s post-hoc test (*p* < 0.05). The groups that do not share the same letter had significantly different results.

We evaluated the activity levels of CYP1A2, CYP2C9, CYP2C19, CYP2D6, CYP3A4, UGT1A1, and sulfotransferases (SULTs) in the CYPs–UGT1A1 KI-HepG2 cells. The CYP1A2, CYP2C9, CYP2C19, CYP2D6, CYP3A4, UGT1A1, and SULTs activities were examined by quantifying acetaminophen, 4′-hydroxy diclofenac, 4′-hydroxy mephenytoin, 1′-hydroxy bufuralol, 1′-hydroxy midazolam, 7′-hydroxy coumarin glucuronide, and 7′-hydroxy coumarin sulfate, respectively. We used the PHHs 48 h as positive controls. The CYP1A2, CYP2C9, CYP2C19, CYP2D6, CYP3A4, and UGT1A1 activity levels in the CYPs–UGT1A1 KI-HepG2 cells were much higher than those in the WT-HepG2 cells (Figure 3 and Figure S2A). The SULT activity levels in the CYPs–UGT1A1 KI-HepG2 cells were similar to those in the WT-HepG2 cells (Figure S2B). In the CYPs–UGT1A1 KI-HepG2 cells, there was no decrease in the activity levels of CYP1A2, CYP2C9, CYP2C19, CYP2D6, CYP3A4, and UGT1A1 during passages 38 to 57 (Figure S3).

**Figure 4:**
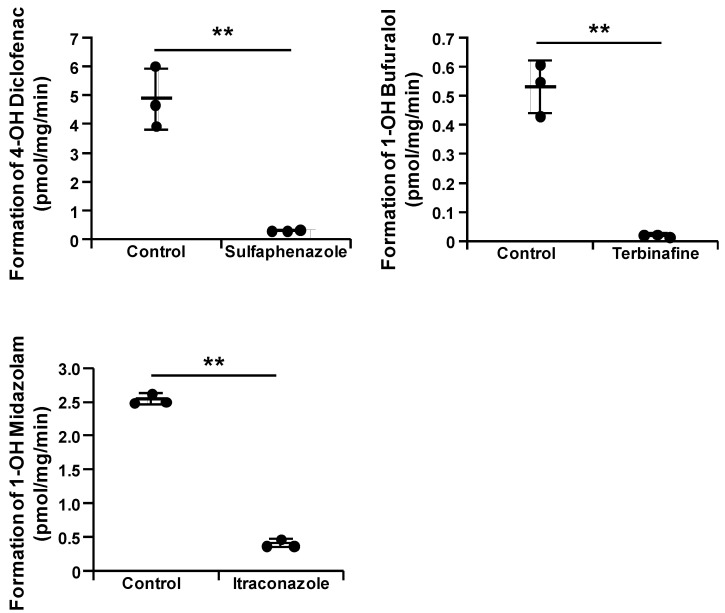
**CYPs-UGT1A1 KI-HepG2 cells can be utilized in predicting drug-drug interactions.** The CYP2C9, CYP2D6, and CYP3A4 activities in the 10 μM sulfaphenazole (a CYP2C9 inhibitor), 10 μM terbinafine (a CYP2D6 inhibitor), or 10 μM itraconazole (a CYP3A4 inhibitor)-treated CYPs–UGT1A1 KI-HepG2 cells were evaluated by quantifying the metabolites of CYP substrates (10 μM diclofenac, 1 μM bufuralol, 10 μM midazolam; these compounds are substrates for CYP2C9, 2D6, and 3A4, respectively). The quantity of metabolites (4′-hydroxy diclofenac, 1′-hydroxy bufuralol, and 1′-hydroxy midazolam; these compounds are metabolites for CYP2C9, 2D6, and 3A4, respectively) were measured by HPLC. Data represent the means ± SD (*n* = 3, technical replicates). Statistical analyses were performed using the unpaired two-tailed Student’s *t* test (** *p* < 0.01).

To examine whether the CYPs–UGT1A1 KI-HepG2 cells can be utilized in drug–drug interaction studies, we treated them with sulfaphenazole (a CYP2C9 inhibitor), terbinafine (a CYP2D6 inhibitor), and itraconazole (a CYP3A4 inhibitor). In the presence of these CYP2C9, CYP2D6, and CYP3A4 inhibitors, the CYP2C9, CYP2D6, and CYP3A4 activities in the CYPs–UGT1A1 KI-HepG2 cells were significantly decreased (Figure 4). Furthermore, sulfaphenazole, terbinafine, and itraconazole were used to calculate the IC50 values in the CYPs–UGT1A1 KI-HepG2 cells. The IC50 values were 0.262 μM, 0.0635 μM, and 0.217 μM for sulfaphenazole, terbinafine, and itraconazole, respectively (Figure S4). The IC50 values of sulfaphenazole, terbinafine, and itraconazole in the CYPs–UGT1A1 KI-HepG2 cells were similar to those previously reported for human liver samples [29,30,31].

**Figure 5:**
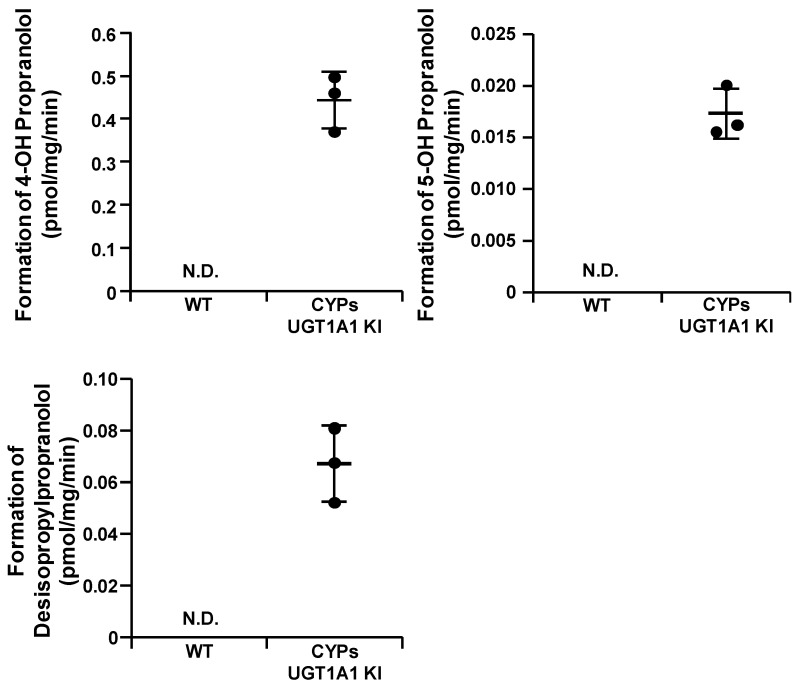
**Major metabolites of propranolol formed by the CYPs–UGT1A1 KI-HepG2 cells.** The CYP1A2 and CYP2D6 activities in the WT-HepG2 cells and the CYPs–UGT1A1 KI-HepG2 cells were evaluated by quantifying the metabolites of 1 μM propranolol. The quantity of major metabolites (4′-hydroxy propranolol, 5′-hydroxy propranolol, and desisopropylpropranolol: metabolites for CYP2D6, 2D6, and 1A2, respectively) were measured by HPLC. Data are means ± SD of three technical replicates. N.D., not detected.

Many drugs are metabolized by multiple cytochrome P450s or in different ways by the same P450. We investigated the metabolism of propranolol in the CYPs–UGT1A1 KI-HepG2 cells. In the WT-HepG2 cells, the three main metabolites (4′-hydroxy propranolol, 5′-hydroxy propranolol, and desisopropylpropranolol) were undetectable, whereas they were present at detectable levels in the CYPs–UGT1A1 KI-HepG2 cells (Figure 5).

These results suggest that the CYPs–UGT1A1 KI-HepG2 cells expressed functional CYP1A2, CYP2C9, CYP2C19, CYP2D6, CYP3A4, and UGT1A1. In addition, the results demonstrated that CYPs–UGT1A1 KI-HepG2 cells can be utilized in drug–drug interaction studies. Moreover, the CYPs–UGT1A1 KI-HepG2 cells were able to predict the metabolites of the drugs that undergo different reactions under the influence of multiple cytochrome P450s.

### 3.3. Prediction of Drug-Induced Liver Injury Using CYPs–UGT1A1 KI-HepG2 Cells

**Figure 6:**
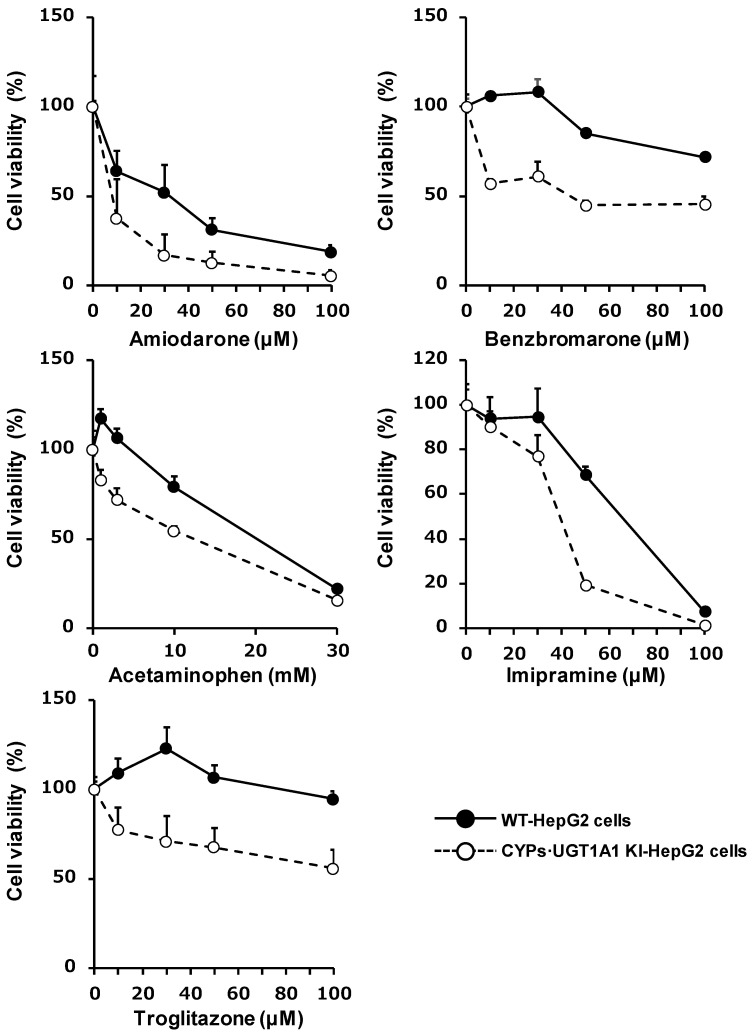
**Drug-induced liver injury in the WT-HepG2 cells and the CYPs–UGT1A1 KI-HepG2 cells.** The WT-HepG2 cells and the CYPs–UGT1A1 KI-HepG2 cells were treated with various concentrations of amiodarone, benzbromarone, acetaminophen, imipramine, or troglitazone. After 24 h, cell viability was examined by WST-8 assay. The cell viability was calculated as a percentage of the number of cells after treatment with a solvent only. The results shown are means ± SD of three technical replicates.

To examine whether CYPs–UGT1A1 KI-HepG2 cells could be used to predict drug-induced liver injury, the WT-HepG2 cells and the CYPs–UGT1A1 KI-HepG2 cells were treated with five hepatotoxic drugs (amiodarone, benzbromarone, acetaminophen, imipramine, and troglitazone), and cell viability was measured by WST-8 assay. We found that the viability of the CYPs–UGT1A1 KI-HepG2 cells was lower than the viability of the WT-HepG2 cells (Figure 6). These results suggest that the CYPs–UGT1A1 KI-HepG2 cells were more sensitive to drug-induced liver toxicity than the WT-HepG2 cells.

## 4. Discussion

We succeeded in generating CYPs–UGT1A1 KI-HepG2 cells using the PITCh system. Furthermore, the CYPs–UGT1A1 KI-HepG2 cells showed drug-metabolizing enzyme gene expression levels comparable to those of PHHs 48 h and had a very high drug-metabolizing capacity compared with the WT-HepG2 cells (Figure 2 and Figure 3). Several other groups have also reported attempts to increase the expression of drug-metabolizing enzymes in HepG2 cells by various methods. Most of them are reports of overexpression of single drug-metabolizing enzymes using plasmid vectors or viral vectors [17,32,33,34,35]. Recently, Kazuki et al. used an artificial chromosome technology to generate HepG2 cells stably expressing CYP2C9, CYP2C19, CYP2D6, CYP3A4, and POR (transchromosomic HepG2 cells) [36]. Consistent with the results of their study, we showed that CYPs–UGT1A1 KI-HepG2 cells can accurately predict drug metabolism and hepatotoxicity. However, transchromosomic HepG2 cells express very little CYP1A2 and UGT1A1, so it is difficult to predict the metabolism of certain drugs such as propranolol (Figure 5). In the metabolism of propranolol, CYP2D6 and CYP1A2 play critical roles and are involved in three main metabolic reactions, yielding 4′-hydroxy propranolol (CYP2D6), 5′-hydroxy propranolol (CYP2D6), and desisopropylpropranolol (CYP1A2), respectively [37,38,39]. Many drugs are excreted from the body after oxidation by cytochrome P450 (phase I reaction) and conjugation via UGT (phase II reaction) [4]. It is necessary to investigate whether CYPs–UGT1A1 KI-HepG2 cells can predict the effects of metabolism by CYPs and subsequent conjugation reactions.

The expression levels of UGT1A1 in the CYPs–UGT1A1 KI-HepG2 cells were comparable to those of PHHs 48 h, but its activity was lower than that of PHHs 48 h (Figure 2 and Figure 3). It is known that 7′-hydroxy coumarin is glucuronidated not only by UGT1A1, but also by various UGT1A isoforms [40,41,42]. Therefore, if multiple UGT isoforms can be overexpressed, it will be possible to generate a model with a UGT metabolic capacity comparable to that of PHHs.

There are multiple SNPs in cytochrome P450 and UGT1A1. SNPs, which can drastically change drug metabolism, may also cause unexpected side effects [43,44]. If the potential effects of individual differences in SNPs could be predicted at early stages of drug discovery research, it would be possible to develop drugs more efficiently. However, most PHH donors used in general drug discovery research are Caucasian, so safety studies reflecting Japanese-specific SNPs such as UGT1A1*6 and CYP2D6*10 cannot be adequately conducted. We have successfully expressed UGT1A1*6 in Caco-2 cells using the PITCh system [24]. Furthermore, we have shown that UGT1A1*6-expressing Caco-2 cells can be applied to drug metabolism and drug-induced toxicity experiments that reflect the effects of patients with UGT1A1*6 [24]. By generating CYPs–UGT1A1 KI-HepG2 cells reflecting the effects of patients with CYP2D6*10 and UGT1A1*6, it will be possible to construct an in vitro hepatocyte model that can predict liver metabolism and toxicity reflecting a more representative range of individual differences.

The CYPs–UGT1A1 KI-HepG2 cells were more sensitive to drug-induced liver toxicity than the WT-HepG2 cells (Figure 6). Amiodarone is metabolized by CYP3A4 to produce desethyl amiodarone, which is known to be more hepatotoxic than amiodarone [45,46]. Benzbromarone is metabolized by CYP2C9 and CYP3A4, and its metabolites have been reported to be hepatotoxic [47,48]. N-acetyl-p-benzoquinone imine (NAPQI), a major metabolite and toxicant of acetaminophen, is reported to be produced not only by CYP2E1, but also by CYP3A4 and CYP1A2 [49,50]. It has also been reported that acetaminophen-induced hepatotoxicity is attenuated in CYP3A4-knockout human iPS cell-derived hepatocyte-like cells [51]. Thus, increased NAPQI levels due to CYP3A4 or CYP1A2 in CYPs–UGT1A1 KI-HepG2 cells may have reduced cell viability. Since CYPs–UGT1A1 KI-HepG2 cells have a higher drug-metabolizing capacity than WT-HepG2 cells (Figure 3), they are a better model to evaluate the potential toxicity of metabolites.

Preclinical drug screening should assess the induction of drug-metabolizing enzymes [6,50,52], which would be difficult to predict because CYPs–UGT1A1 KI-HepG2 cells overexpress CYPs and UGT1A1 using the CAG promoter. Therefore, preclinical drug screening using CYPs–UGT1A1 KI-HepG2 cells would be limited to drug metabolism, drug–drug interactions, and drug-induced liver toxicity.

## 5. Conclusions

Using the PITCh system, we successfully generated CYPs–UGT1A1 KI-HepG2 cells. The CYPs–UGT1A1 KI-HepG2 cells expressed functional CYPs and UGT1A1. Furthermore, the CYPs–UGT1A1 KI-HepG2 cells showed use for predicting drug metabolism, drug–drug interactions, and drug-induced liver toxicity that cannot be assessed using single drug-metabolizing enzyme overexpression models. CYPs–UGT1A1 KI-HepG2 cells will be a helpful new hepatocyte model for drug discovery research.

## Data Availability

The authors declare that all the data related to this study are available within the paper or can be obtained from the authors upon reasonable request.

## Supplementary Materials

The following supporting information can be downloaded at https://www.mdpi.com/article/10.3390/cells11101677/s1, Figure S1: Expression analysis of drug-metabolizing enzymes in different passage numbers of CYPs–UGT1A1 KI-HepG2 cells; Figure S2: UGT1A1 and SULTs activity evaluation of the CYPs–UGT1A1 KI-HepG2 cells; Figure S3: Evaluation of drug-metabolizing activity in different passage numbers of CYPs–UGT1A1 KI-HepG2 cells; Figure S4: IC50 of sulfaphenazole, terbinafine, and itraconazole in the CYPs–UGT1A1 KI-HepG2 cells; Table S1: HPLC methods; Supplementary Sequences S1: Sequences of the donor plasmids used in this study.

## References

[1] Godoy P., Hewitt N.J., Albrecht U., Andersen M.E., Ansari N., Bhattacharya S., Bode J.G., Bolleyn J., Borner C., Böttger J. (2013). Recent advances in 2D and 3D in vitro systems using primary hepatocytes, alternative hepatocyte sources and non-parenchymal liver cells and their use in investigating mechanisms of hepatotoxicity, cell signaling and ADME. Arch. Toxicol..

[2] Qiao S., Feng S., Wu Z., He T., Ma C., Peng Z., Tian E., Pan G. (2021). Functional Proliferating Human Hepatocytes: In Vitro Hepatocyte Model for Drug Metabolism, Excretion, and Toxicity. Drug Metab. Dispos..

[3] Hoofnagle J.H., Björnsson E.S. (2019). Drug-Induced Liver Injury—Types and Phenotypes. N. Engl. J. Med..

[4] Guengerich F.P. (2008). Cytochrome p450 and chemical toxicology. Chem. Res. Toxicol..

[5] Williams J.A., Hyland R., Jones B.C., Smith D.A., Hurst S., Goosen T.C., Peterkin V., Koup J.R., Ball S.E. (2004). Drug-drug interactions for UDP-glucuronosyltransferase substrates: A pharmacokinetic explanation for typically observed low exposure (AUCi/AUC) ratios. Drug Metab. Dispos..

[6] Lin J.H., Lu A.Y. (1998). Inhibition and induction of cytochrome P450 and the clinical implications. Clin. Pharmacokinet..

[7] Agrawal V., Choi J.H., Giacomini K.M., Miller W.L. (2010). Substrate-specific modulation of CYP3A4 activity by genetic variants of cytochrome P450 oxidoreductase. Pharmacogenet. Genom..

[8] Weaver R.J., Blomme E.A., Chadwick A.E., Copple I.M., Gerets H.H.J., Goldring C.E., Guillouzo A., Hewitt P.G., Ingelman-Sundberg M., Jensen K.G. (2020). Managing the challenge of drug-induced liver injury: A roadmap for the development and deployment of preclinical predictive models. Nat. Rev. Drug Discov..

[9] Yokoyama Y., Sasaki Y., Terasaki N., Kawataki T., Takekawa K., Iwase Y., Shimizu T., Sanoh S., Ohta S. (2018). Comparison of Drug Metabolism and Its Related Hepatotoxic Effects in HepaRG, Cryopreserved Human Hepatocytes, and HepG2 Cell Cultures. Biol. Pharm. Bull..

[10] Brandon E.F.A., Raap C.D., Meijerman I., Beijnen J.H., Schellens J.H.M. (2003). An update on in vitro test methods in human hepatic drug biotransformation research: Pros and cons. Toxicol. Appl. Pharmacol..

[11] Schulz C., Kammerer S., Küpper J.-H. (2019). NADPH-cytochrome P450 reductase expression and enzymatic activity in primary-like human hepatocytes and HepG2 cells for in vitro biotransformation studies. Clin. Hemorheol. Microcirc..

[12] Hu H., Gehart H., Artegiani B., LÖpez-Iglesias C., Dekkers F., Basak O., van Es J., Chuva de Sousa Lopes S.M., Begthel H., Korving J. (2018). Long-Term Expansion of Functional Mouse and Human Hepatocytes as 3D Organoids. Cell.

[13] Peng W.C., Logan C.Y., Fish M., Anbarchian T., Aguisanda F., Álvarez-Varela A., Wu P., Jin Y., Zhu J., Li B. (2018). Inflammatory Cytokine TNFα Promotes the Long-Term Expansion of Primary Hepatocytes in 3D Culture. Cell.

[14] Hendriks D., Artegiani B., Hu H., Chuva de Sousa Lopes S., Clevers H. (2021). Establishment of human fetal hepatocyte organoids and CRISPR-Cas9-based gene knockin and knockout in organoid cultures from human liver. Nat. Protoc..

[15] Westerink W.M.A., Schoonen W.G.E.J. (2007). Cytochrome P450 enzyme levels in HepG2 cells and cryopreserved primary human hepatocytes and their induction in HepG2 cells. Toxicol. Vitr..

[16] Westerink W.M.A., Schoonen W.G.E.J. (2007). Phase II enzyme levels in HepG2 cells and cryopreserved primary human hepatocytes and their induction in HepG2 cells. Toxicol. Vitr..

[17] Chen S., Wu Q., Li X., Li D., Mei N., Ning B., Puig M., Ren Z., Tolleson W.H., Guo L. (2021). Characterization of cytochrome P450s (CYP)-overexpressing HepG2 cells for assessing drug and chemical-induced liver toxicity. J. Environ. Sci. Health Part C Toxicol. Carcinog..

[18] Takayama K., Mizuguchi H. (2017). Generation of human pluripotent stem cell-derived hepatocyte-like cells for drug toxicity screening. Drug Metab. Pharmacokinet..

[19] Takayama K., Morisaki Y., Kuno S., Nagamoto Y., Harada K., Furukawa N., Ohtaka M., Nishimura K., Imagawa K., Sakurai F. (2014). Prediction of interindividual differences in hepatic functions and drug sensitivity by using human iPS-derived hepatocytes. Proc. Natl. Acad. Sci. USA.

[20] Deguchi S., Yamashita T., Igai K., Harada K., Toba Y., Hirata K., Takayama K., Mizuguchi H. (2019). Modeling of Hepatic Drug Metabolism and Responses in CYP2C19 Poor Metabolizer Using Genetically Manipulated Human iPS cells. Drug Metab. Dispos..

[21] Baxter M., Withey S., Harrison S., Segeritz C.-P., Zhang F., Atkinson-Dell R., Rowe C., Gerrard D.T., Sison-Young R., Jenkins R. (2015). Phenotypic and functional analyses show stem cell-derived hepatocyte-like cells better mimic fetal rather than adult hepatocytes. J. Hepatol..

[22] Kratochwil N.A., Meille C., Fowler S., Klammers F., Ekiciler A., Molitor B., Simon S., Walter I., McGinnis C., Walther J. (2017). Metabolic Profiling of Human Long-Term Liver Models and Hepatic Clearance Predictions from In Vitro Data Using Nonlinear Mixed-Effects Modeling. AAPS J..

[23] Nghiem-Rao T.H., Pfeifer C., Asuncion M., Nord J., Schill D., Pulakanti K., Patel S.B., Cirillo L.A., Rao S. (2021). Human induced pluripotent stem cell derived hepatocytes provide insights on parenteral nutrition associated cholestasis in the immature liver. Sci. Rep..

[24] Negoro R., Yamada N., Watanabe K., Kono Y., Fujita T. (2022). Generation of Caco-2 cells stably expressing CYP3A4·POR·UGT1A1 and CYP3A4·POR·UGT1A1*6 using a PITCh system. Arch. Toxicol..

[25] Deguchi S., Tsuda M., Kosugi K., Sakamoto A., Mimura N., Negoro R., Sano E., Nobe T., Maeda K., Kusuhara H. (2021). Usability of Polydimethylsiloxane-Based Microfluidic Devices in Pharmaceutical Research Using Human Hepatocytes. ACS Biomater. Sci. Eng..

[26] Sano E., Deguchi S., Matsuoka N., Tsuda M., Wang M., Kosugi K., Mori C., Yagi K., Wada A., Yamasaki S. (2021). Generation of Tetrafluoroethylene-Propylene Elastomer-Based Microfluidic Devices for Drug Toxicity and Metabolism Studies. ACS Omega.

[27] Sakuma T., Nishikawa A., Kume S., Chayama K., Yamamoto T. (2014). Multiplex genome engineering in human cells using all-in-one CRISPR/Cas9 vector system. Sci. Rep..

[28] Sakuma T., Nakade S., Sakane Y., Suzuki K.I.T., Yamamoto T. (2016). MMEJ-Assisted gene knock-in using TALENs and CRISPR-Cas9 with the PITCh systems. Nat. Protoc..

[29] Shirasaka Y., Chang S.-Y., Grubb M.F., Peng C.-C., Thummel K.E., Isoherranen N., Rodrigues A.D. (2013). Effect of CYP3A5 expression on the inhibition of CYP3A-catalyzed drug metabolism: Impact on modeling CYP3A-mediated drug-drug interactions. Drug Metab. Dispos..

[30] McGinnity D.F., Tucker J., Trigg S., Riley R.J. (2005). Prediction of CYP2C9-mediated drug-drug interactions: A comparison using data from recombinant enzymes and human hepatocytes. Drug Metab. Dispos..

[31] Bell L., Bickford S., Nguyen P.H., Wang J., He T., Zhang B., Friche Y., Zimmerlin A., Urban L., Bojanic D. (2008). Evaluation of fluorescence- and mass spectrometry-based CYP inhibition assays for use in drug discovery. J. Biomol. Screen..

[32] Hosomi H., Fukami T., Iwamura A., Nakajima M., Yokoi T. (2011). Development of a highly sensitive cytotoxicity assay system for CYP3A4-mediated metabolic activation. Drug Metab. Dispos..

[33] Steinbrecht S., Pfeifer N., Herzog N., Katzenberger N., Schulz C., Kammerer S., Küpper J.-H. (2020). HepG2-1A2 C2 and C7: Lentivirus vector-mediated stable and functional overexpression of cytochrome P450 1A2 in human hepatoblastoma cells. Toxicol. Lett..

[34] Yoshitomi S., Ikemoto K., Takahashi J., Miki H., Namba M., Asahi S. (2001). Establishment of the transformants expressing human cytochrome P450 subtypes in HepG2, and their applications on drug metabolism and toxicology. Toxicol. Vitr..

[35] Xuan J., Chen S., Ning B., Tolleson W.H., Guo L. (2016). Development of HepG2-derived cells expressing cytochrome P450s for assessing metabolism-associated drug-induced liver toxicity. Chem. Biol. Interact..

[36] Satoh D., Iwado S., Abe S., Kazuki K., Wakuri S., Oshimura M., Kazuki Y. (2017). Establishment of a novel hepatocyte model that expresses four cytochrome P450 genes stably via mammalian-derived artificial chromosome for pharmacokinetics and toxicity studies. PLoS ONE.

[37] Otey C.R., Bandara G., Lalonde J., Takahashi K., Arnold F.H. (2006). Preparation of human metabolites of propranolol using laboratory-evolved bacterial cytochromes P450. Biotechnol. Bioeng..

[38] Walle T., Walle U.K., Olanoff L.S. (1985). Quantitative account of propranolol metabolism in urine of normal man. Drug Metab. Dispos..

[39] Yoshimoto K., Echizen H., Chiba K., Tani M., Ishizaki T. (1995). Identification of human CYP isoforms involved in the metabolism of propranolol enantiomers--N-desisopropylation is mediated mainly by CYP1A2. Br. J. Clin. Pharmacol..

[40] Cheng Z., Radominska-Pandya A., Tephly T.R. (1999). Studies on the substrate specificity of human intestinal UDP-lucuronosyltransferases 1A8 and 1A10. Drug Metab. Dispos..

[41] Juvonen R.O., Heikkinen A.T., Kärkkäinen O., Jehangir R., Huuskonen J., Troberg J., Raunio H., Pentikäinen O.T., Finel M. (2020). In vitro glucuronidation of 7-hydroxycoumarin derivatives in intestine and liver microsomes of Beagle dogs. Eur. J. Pharm. Sci..

[42] Juvonen R.O., Rauhamäki S., Kortet S., Niinivehmas S., Troberg J., Petsalo A., Huuskonen J., Raunio H., Finel M., Pentikäinen O.T. (2018). Molecular Docking-Based Design and Development of a Highly Selective Probe Substrate for UDP-glucuronosyltransferase 1A10. Mol. Pharm..

[43] Ingelman-Sundberg M. (2001). Genetic susceptibility to adverse effects of drugs and environmental toxicants: The role of the CYP family of enzymes. Mutat. Res.-Fundam. Mol. Mech. Mutagen..

[44] Sai K., Saeki M., Saito Y., Ozawa S., Katori N., Jinno H., Hasegawa R., Kaniwa N., Sawada J., Komamura K. (2004). UGT1A1 haplotypes associated with reduced glucuronidation and increased serum bilirubin in irinotecan-administered Japanese patients with cancer. Clin. Pharmacol. Ther..

[45] Zahno A., Brecht K., Morand R., Maseneni S., Török M., Lindinger P.W., Krähenbühl S. (2011). The role of CYP3A4 in amiodarone-associated toxicity on HepG2 cells. Biochem. Pharmacol..

[46] Shayeganpour A., El-Kadi A.O.S., Brocks D.R. (2006). Determination of the enzyme(s) involved in the metabolism of amiodarone in liver and intestine of rat: The contribution of cytochrome P450 3A isoforms. Drug Metab. Dispos..

[47] Kitagawara Y., Ohe T., Tachibana K., Takahashi K., Nakamura S., Mashino T. (2015). Novel Bioactivation Pathway of Benzbromarone Mediated by Cytochrome P450. Drug Metab. Dispos..

[48] Kobayashi K., Kajiwara E., Ishikawa M., Mimura H., Oka H., Ejiri Y., Hosoda M., Chiba K. (2013). Cytotoxic effects of benzbromarone and its 1′-hydroxy metabolite in human hepatocarcinoma FLC4 cells cultured on micro-space cell culture plates. Drug Metab. Pharmacokinet..

[49] James L.P., Mayeux P.R., Hinson J.A. (2003). Acetaminophen-induced hepatotoxicity. Drug Metab. Dispos..

[50] Guengerich F.P. (2021). A history of the roles of cytochrome P450 enzymes in the toxicity of drugs. Toxicol. Res..

[51] Deguchi S., Shintani T., Harada K., Okamoto T., Takemura A., Hirata K., Ito K., Takayama K., Mizuguchi H. (2021). In Vitro Model for a Drug Assessment of Cytochrome P450 Family 3 Subfamily A Member 4 Substrates Using Human Induced Pluripotent Stem Cells and Genome Editing Technology. Hepatol. Commun..

[52] Pelkonen O., Turpeinen M., Hakkola J., Honkakoski P., Hukkanen J., Raunio H. (2008). Inhibition and induction of human cytochrome P450 enzymes: Current status. Arch. Toxicol..

