# Supplementary material for: Generation of HepG2 Cells with High Expression of Multiple Drug-Metabolizing Enzymes for Drug Discovery Research Using a PITCh System
Source: Cells. 2022 May 18;11(10):1677. doi: 10.3390/cells11101677 (PMC9140068; doi:10.3390/cells11101677)
Supplement: Supplementary file 1 [file cells-11-01677-s001.zip › cells-1651519-supplementary.pdf]

Supplemental Information

Title

Generation of HepG2 cells with high expression of multiple drug-metabolizing enzymes for drug discovery research using a PITCh system

Authors

Ryosuke Negoro, Mitsuki Tasaka, Sayaka Deguchi, Kazuo Takayama, Takuya Fujita

Supplemental Figures

Figure S1. Expression analysis of drug-metabolizing enzymes in different passage numbers of CYPs-UGT1A1 KI-HepG2 cells, Related to Figure 2.

Figure S2. UGTs and SULTs activity evaluation of CYPs-UGT1A1 KI-HepG2 cells, Related to Figure 3.

Figure S3. Evaluation of drug-metabolizing activity in different passage numbers of CYPs-UGT1A1 KI-HepG2 cells, Related to Figure 3.

Figure S4. IC50 of sulfaphenazole, terbinafine and itraconazole in CYPs-UGT1A1 KI-HepG2 cells, Related to Figure 4.

Supplemental Tables

Table S1. The HPLC methods

Supplemental Experimental Procedures

Supplementary Sequences

Supplementary Sequences S1. Sequences of the donor plasmids used in this study

Supplemental Figures

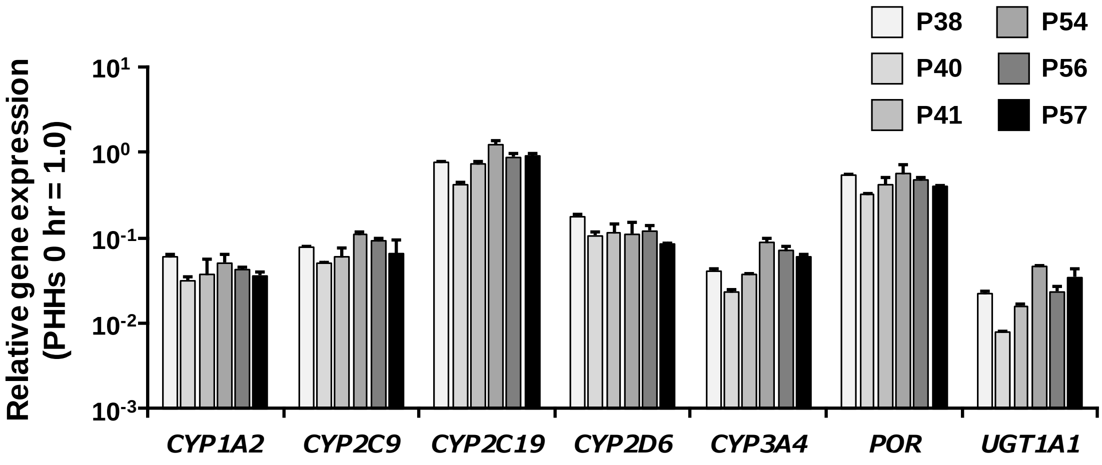

Figure S1. Expression analysis of drug-metabolizing enzymes in different passage numbers of CYPs-UGT1A1 KI-HepG2 cells.

Real time RT-PCR was used to determine expression levels of genes for the drug-metabolizing enzymes *cytochrome P450 family 1 subfamily A member 2* (*CYP1A2*), *cytochrome P450 family 2 subfamily C member 9* (*CYP2C9*), *cytochrome P450 family 2 subfamily C member 19* (*CYP2C19*), *cytochrome P450 family 2 subfamily D member 6* (*CYP2D6*), *cytochrome P450 family 3 subfamily A member 4* (*CYP3A4*), *P450 oxidoreductase* (*POR*), and *UDP glucuronosyltransferase family 1 member A1* (*UGT1A1*) in CYPs-UGT1A1 KI-HepG2 cells (passage number 38, 40, 41, 54, 56 and 57). On the y axis, the gene expression levels in the PHHs 0 hr were taken as 1.0. Data represent the means ± SDs (*n*=3, technical replicates).

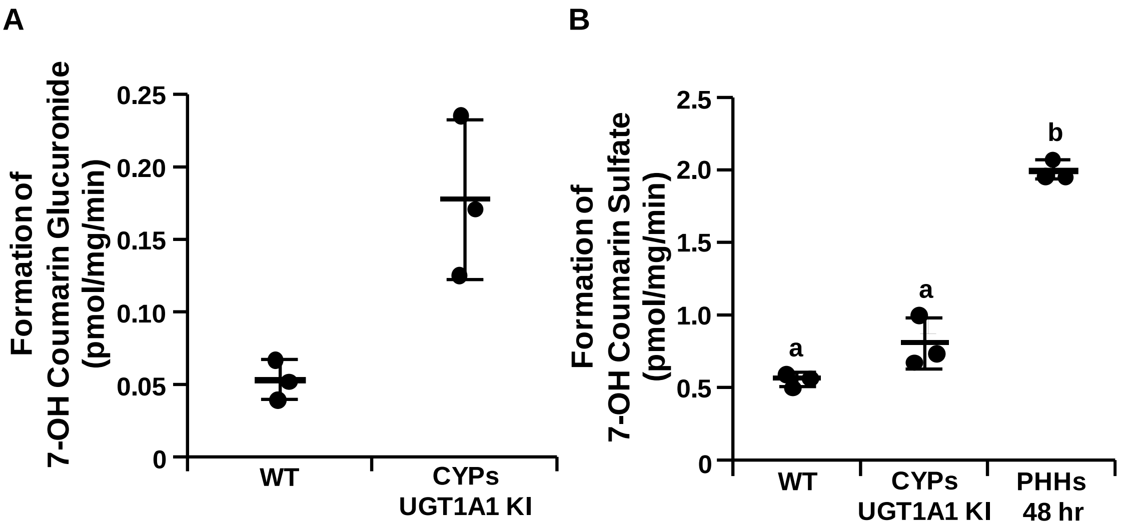

Figure S2. UGT1A1 and SULTs activity evaluation of CYPs-UGT1A1 KI-HepG2 cells.

(A) The UGT1A1 activities in WT-HepG2 cells and CYPs-UGT1A1 KI-HepG2 cells were examined by quantifying the metabolites of 7'-hydroxy coumarin glucuronide (metabolites for UGTs). The quantity of 7'-hydroxy coumarin glucuronide was measured by HPLC. Data represent the means ± SDs (*n*=3, technical replicates). (B) The sulfotransferases (SULTs) activities in WT-HepG2 cells, CYPs-UGT1A1 KI-HepG2 cells and 48 hours cultured primary human hepatocytes (PHHs 48 hr) were examined by quantifying the metabolites of 7'-hydroxy coumarin sulfate (metabolites for SULTs). The quantity of 7'-hydroxy coumarin sulfate was measured by HPLC. Data represent the means ± SDs (*n*=3, technical replicates). Statistical significance was evaluated by one-way ANOVA followed by Tukey's post hoc test ($p < 0.05$). Groups that do not share the same letter are significantly different from each other.

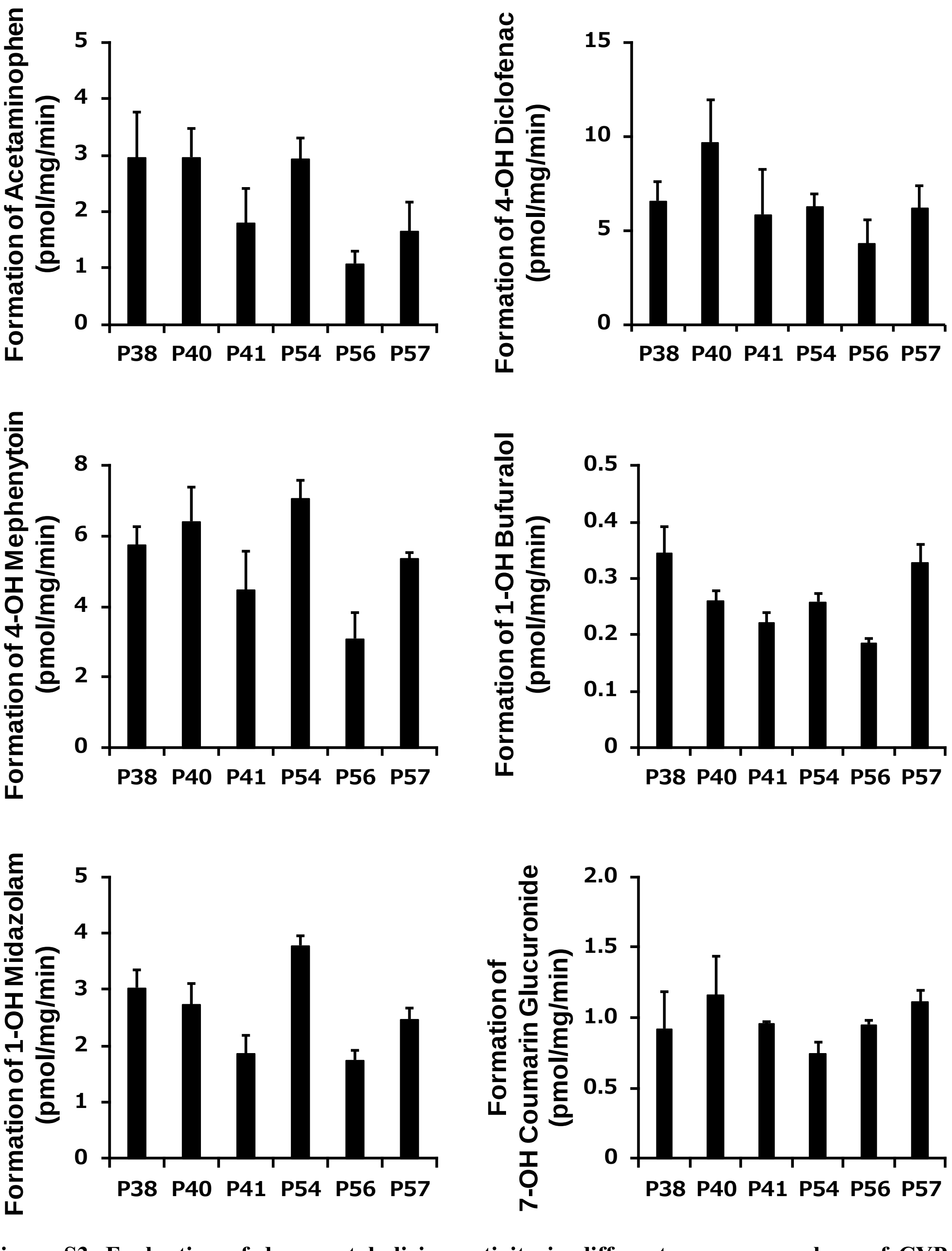

Figure S3. Evaluation of drug-metabolizing activity in different passage numbers of CYPs-UGT1A1 KI-HepG2 cells.

The CYP1A2, CYP2C9, CYP2C19, CYP2D6, CYP3A4 and UGT1A1 activities in CYPs-UGT1A1 KI-HepG2 cells (passage number 38, 40, 41, 54, 56 and 57) were examined by quantifying the metabolites of CYP and UGT substrates (10 μM phenacetin, 10 μM diclofenac, 50 μM S-mephenytoin, 1 μM bufuralol, 10 μM midazolam and 10 μM 7'-hydroxy coumarin; these compounds

are substrates for CYP1A2, 2C9, 2C19, 2D6, 3A4 and UGTs, respectively). The quantity of metabolites (acetaminophen, 4'-hydroxy diclofenac, 4'-hydroxy mephenytoin, 1'-hydroxy bufuralol, 1'-hydroxy midazolam and 7'-hydroxy coumarin glucuronide; these compounds are metabolites for CYP1A2, 2C9, 2C19, 2D6, 3A4 and UGTs, respectively) were measured by HPLC. Data represent the means ± SDs (n = 3, technical replicates).

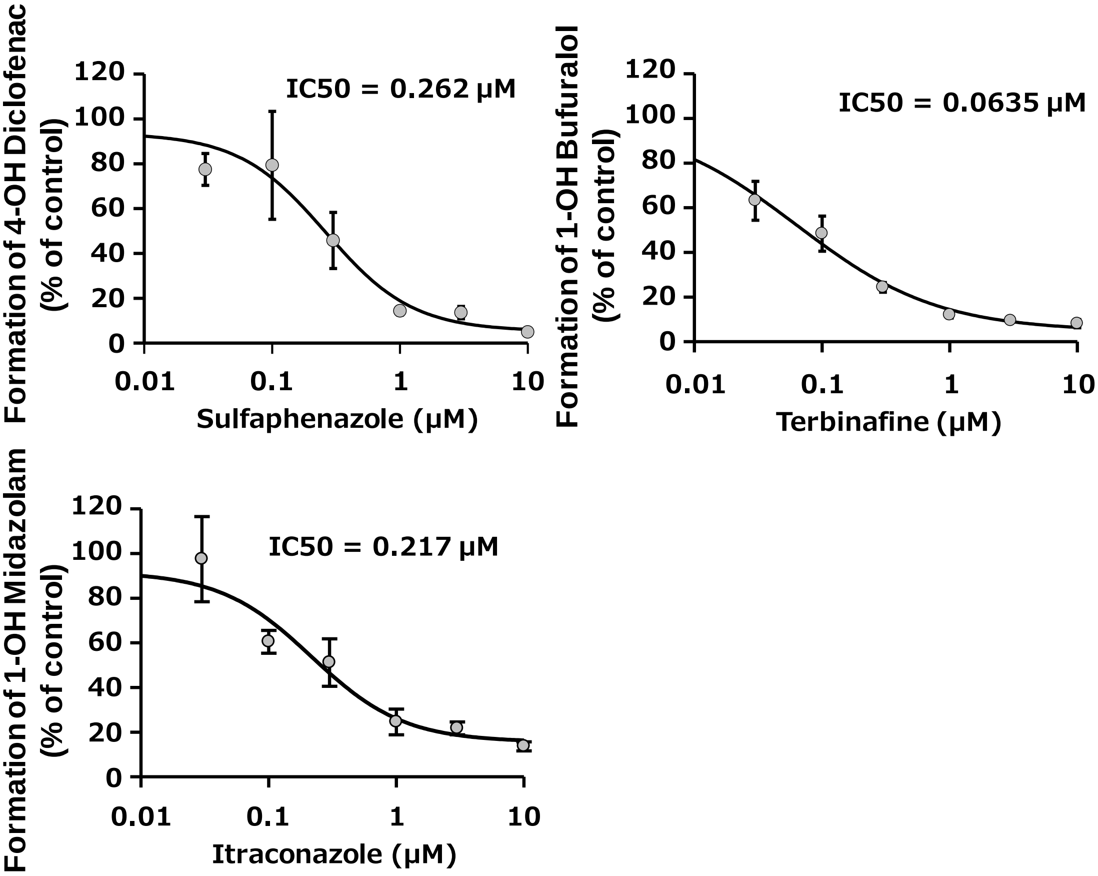

Figure S4. IC50 of sulfaphenazole, terbinafine and itraconazole in CYPs-UGT1A1 KI-HepG2 cells.

The CYP2C9, CYP2D6 and CYP3A4 activities in 0.03-10 μM sulfaphenazole (a CYP2C9 inhibitor), 0.03-10 μM terbinafine (a CYP2D6 inhibitor) or 0.03-10 μM itraconazole (a CYP3A4 inhibitor)-treated CYPs-UGT1A1 KI-HepG2 cells were evaluated by quantifying the metabolites of CYP substrates (10 μM diclofenac, 1 μM bufuralol, 10 μM midazolam; these compounds are substrates for CYP2C9,2D6 and 3A4, respectively). The quantity of metabolites (4'-hydroxy diclofenac, 1'-hydroxy bufuralol and 1'-hydroxy midazolam; these compounds are metabolites for CYP2C9, 2D6 and 3A4, respectively) were measured by HPLC. Data represent the mean ± SD (*n*=3, technical replicates).

Supplementary Table S1. The HPLC methods

**Acetaminophen**	
column	COSMOSIL(R) 5C18-AR-II<br>Packed Column 4.6 mm I.D. x 150 mm<br>(Nacalai Tesque, #38144-31)
Mobile phase (A)	20 mM Phosphate buffer (pH 3.0)
Mobile phase (B)	Acetonitrile
Flow-rate (ml/min)	1 ml/min
Time (min)	Mobile phase A (%) : Mobile phase B (%)
0	90 : 10
1.5	90 : 10
6	0 : 100
7	0 : 100
7.01	90 : 10
12	90 : 10
Temperature	40 ℃
Detection	UV 244 nm
Retention time	3.8 min

**4’-hydroxy diclofenac**	
column	COSMOSIL(R) 5C18-AR-II<br>Packed Column 4.6 mm I.D. x 150 mm<br>(Nacalai Tesque, #38144-31)
Mobile phase (A)	20 mM Phosphate buffer (pH 3.0)
Mobile phase (B)	Acetonitrile
Flow-rate (ml/min)	1 ml/min A:B = 55:45, v/v
Temperature	40 ℃
Detection	UV 280 nm
Retention time	4.9 min

**4’-hydroxy mephenytoin**	
column	COSMOSIL(R) 5C18-AR-II<br>Packed Column 4.6 mm I.D. x 150 mm<br>(Nacalai Tesque, #38144-31)
Mobile phase (A)	20 mM Phosphate buffer (pH 3.0)
Mobile phase (B)	Acetonitrile
Flow-rate<br>(ml/min)	1 ml/min
Time (min)	Mobile phase A (%) : Mobile phase B (%)
0	77.5 : 22.5
1.5	77.5 : 22.5
6	0 : 100
7	0 : 100
7.01	77.5 : 22.5
12	77.5 : 22.5
Temperature	35 ℃
Detection	UV 211 nm
Retention time	4.7 min

**1’-hydroxy bufuralol**	
column	COSMOSIL(R) 5C18-AR-II<br>Packed Column 4.6 mm I.D. x 150 mm<br>(Nacalai Tesque, #38144-31)
Mobile phase (A)	20 mM Phosphate buffer (pH 3.0)
Mobile phase (B)	Acetonitrile
Flow-rate (ml/min)	1 ml/min
Time (min)	Mobile phase A (%) : Mobile phase B (%)
0	80 : 20
1.5	80 : 20
6	0 : 100
7	0 : 100
7.01	80 : 20
12	80 : 20
Temperature	40 ℃
Detection	Fluorescence 247 nm / 297 nm
Retention time	3.8 min

**1’-hydroxy midazolam**	
column	COSMOSIL(R) 5C18-MS-II<br>Packed Column 4.6 mm I.D. x 150 mm<br>(Nacalai Tesque, #38019-81)
Mobile phase (A)	10 mM Phosphate buffer (pH 7.4)
Mobile phase (B)	Acetonitrile : Methanol (7:5, v/v)
Flow-rate (ml/min)	1 ml/min A:B = 50:50, v/v
Temperature	40 ℃
Detection	UV 220 nm
Retention time	7.2 min

**7’-hydroxy coumarin glucuronide<br>7’-hydroxy coumarin sulfate**	
column	COSMOSIL(R) 5C18-AR-II<br>Packed Column 4.6 mm I.D. x 150 mm<br>(Nacalai Tesque, #38144-31)
Mobile phase (A)	20 mM Phosphate buffer (pH 3.0)
Mobile phase (B)	Acetonitrile
Flow-rate<br>(ml/min)	1 ml/min A:B = 85:15, v/v
Temperature	40 ℃
Detection	Fluorescence 315 nm / 365 nm
Retention time	7’-hydroxy coumarin glucuronide : 2.8 min<br>7’-hydroxy coumarin sulfate : 4.5 min

**4’-hydroxy propranolol<br>5’-hydroxy propranolol<br>desisopropylpropranolol**	
column	COSMOSIL(R) 5C18-AR-II<br>Packed Column 4.6 mm I.D. x 150 mm<br>(Nacalai Tesque, #38144-31)
Mobile phase (A)	20 mM Phosphate buffer (pH 3.0)
Mobile phase (B)	Acetonitrile
Flow-rate (ml/min)	1 ml/min A:B = 75:25, v/v
Temperature	40 ℃
Detection	Fluorescence 310 nm / 380 nm
Retention time	4’-hydroxy propranolol : 3.4 min<br>5’-hydroxy propranolol : 2.9 min<br>desisopropylpropranolol : 4.6 min

Supplemental Experimental Procedures

CRISPR-Cas9 Plasmid

sgRNA sequences are shown below.

AAVS1 gRNA: gcggctccaattcggaagtg

CCR5 gRNA: tcagtttacacccgatccac

CYP3A7 gRNA : cctgaaattacgctttggag

hROSA26 gRNA : cctcgcgtgatctcgtcatc

Supplementary Sequences S1. Sequences of the donor plasmids used in this study

Donor Plasmid (PITCh AAVS1 pCAG-CYP3A4-P2A-POR-T2A-PuroR-pA)

Full sequences of PITCh AAVS1 pCAG-CYP3A4-P2A-POR-T2A-PuroR-pA donor plasmid is shown below.

PITCh gRNA target site, AAVS1 homology arm, CYP3A4, POR, PuroR

gcatcgtacgcgtacgtgtttgggttgccagtctcgatccgccccgtcgttcctggccctgggctttgccaccctatgctgacaccccgtcccagtc
cccttaccattccccttcgaccaccTAGTTATTAATAGTAATCAATTACGGGGTCATTAGTTCATAGCCCA
TATATGGAGTTCCGCGTTACATAACTTACGGTAAATGGCCCGCCTGGCTGACCGCCCAAC
GACCCCCGCCCATTGACGTCAATAATGACGTATGTTCCCATAGTAACGCCAATAGGGACTT
TCCATTGACGTCAATGGGTGGAGTATTTACGGTAAACTGCCCACTTGGCAGTACATCAAG
TGTATCATATGCCAAGTACGCCCCCTATTGACGTCAATGACGGTAAATGGCCCGCCTGGCA
TTATGCCCAGTACATGACCTTATGGGACTTTCCTACTTGGCAGTACATCTACGTATTAGTCA
TCGCTATTACCATGGTCGAGGTGAGCCCCACGTTCTGCTTCACTCTCCCCATCTCCCCCCC
CTCCCCACCCCCAATTTTGTATTTATTTATTTTTTAATTATTTTGTGCAGCGATGGGGGCGG
GGGGGGGGGGGGGCGCGCGCCAGGCGGGGCGGGGCGGGGCGAGGGGCGGGGCGGG
GCGAGGCGGAGAGGTGCGGCGGCAGCCAATCAGAGCGGCGCGCTCCGAAAGTTTCCTT
TTATGGCGAGGCGGCGGCGGCGGCGGCCCTATAAAAAGCGAAGCGCGCGGCGGGCGGG
AGTCGCTGCGCGCTGCCTTCGCCCCGTGCCCCGCTCCGCCGCCGCCTCGCGCCGCCCGC
CCCGGCTCTGACTGACCGCGTTACTCCCACAGGTGAGCGGGCGGGACGGCCCTTCTCCT
CCGGGCTGTAATTAGCGCTTGGTTTAATGACGGCTTGTTTCTTTTCTGTGGCTGCGTGAAA
GCCTTGAGGGGCTCCGGGAGGGCCCTTTGTGCGGGGGGAGCGGCTCGGGGGGTGCGTG
CGTGTGTGTGTGCGTGGGGAGCGCCGCGTGCGGCTCCGCGCTGCCCGGCGGCTGTGAGC
GCTGCGGGCGCGGCGCGGGGCTTTGTGCGCTCCGCAGTGTGCGCGAGGGGAGCGCGGC
CGGGGGCGGTGCCCCGCGGTGCGGGGGGGGCTGCGAGGGGAACAAAGGCTGCGTGCG
GGGTGTGTGCGTGGGGGGGTGAGCAGGGGGTGTGGGCGCGTCGGTCGGGCTGCAACCC
CCCCTGCACCCCCCTCCCCGAGTTGCTGAGCACGGCCCGGCTTCGGGTGCGGGGCTCCG
TACGGGGCGTGGCGCGGGGCTCGCCGTGCCGGGCGGGGGGTGGCGGCAGGTGGGGGTG
CCGGGCGGGGCGGGGCCGCCTCGGGCCGGGGAGGGCTCGGGGGAGGGGCGCGGCGGC

CCCCGGAGCGCCGGCGGCTGTCGAGGCGCGGCGAGCCGCAGCCATTGCCTTTTATGGTA
ATCGTGCGAGAGGGCGCAGGGACTTCCTTTGTCCCAAATCTGTGCGGAGCCGAAATCTG
GGAGGCGCCGCCGCACCCCCTCTAGCGGGCGCGGGGCGAAGCGGTGCGGCGCCGGCAG
GAAGGAAATGGGCGGGGAGGGCCTTCGTGCGTCGCCGCGCCGCCGTCCCCTTCTCCCTC
TCCAGCCTCGGGGCTGTCCGCGGGGGGACGGCTGCCTTCGGGGGGGACGGGGCAGGGC
GGGGTTCGGCTTCTGGCGTGTGACCGGCGGCTCTAGAGCCTCTGCTAACCATGTTCATGC
CTTCTTCTTTTTCCTACAGCTCCTGGGCAACGTGCTGGTTATTGTGCTGTCTCATCATTTTG
GCAAAGAATTAACCCTCACTAAAGGGGTACCGGGCCCCCCCTCGAGGTCGACGGTATCG
ATAAGCTTGATATCGAATTgccgccatggctctcatcccagacttggccatggaaacctggcttctcctggctgtcagcctggtgc
tcctctatctatatggaacccattcacatggactttttaagaagcttggaattccagggcccacacctctgcctttttgggaaatattttgtcctacca ta
agggcttttgtatgtttgacatggaatgtcataaaaagtatggaaaagtgtggggcttttatgatggtcaacagcctgtgctggctatcacagatcctg
acatgatcaaaacagtgctagtgaaagaatgttattctgtcttcacaaaccggaggccttttggtccagtgggatttatgaaaagtgccatctctatag
ctgaggatgaagaatggaagagattacgatcattgctgtctccaaccttcaccagtggaaaactcaaggagatggtccctatcattgcccagtatg
gagatgtgttggtgagaaatctgaggcgggaagcagagacaggcaagcctgtcaccttgaaagacgtctttggggcctacagcatggatgtgat
cactagcacatcatttggagtgaacatcgactctctcaacaatccacaagacccctttgtggaaaacaccaagaagcttttaagatttgattttttggat
ccattctttctctcaataacagtctttccattcctcatcccaattcttgaagtattaaatatctgtgtgtttccaagagaagttacaaatttttaagaaaatct
gtaaaaaggatgaaagaaagtcgcctcgaagatacacaaaagcaccgagtggatttccttcagctgatgattgactctcagaattcaaaagaaact
gagtcccacaaagctctgtccgatctggagctcgtggcccaatcaattatctttatttttgctggctatgaaaccac gagcagtgttctctccttcattat
gtatgaactggccactcaccctgatgtccagcagaaactgcaggaggaaattgatgcagttttacccaataaggcaccacccacctatgatactgt
gctacagatggagtatcttgacatggtggtgaatgaaacgctcagattattcccaattgctatgagacttgagagggtctgcaaaaaagatgttgag
atcaatgggatgttcattcccaaaggggtggtggtgatgattccaagctatgctcttcaccgtgacccaaagtactggacagagcctgagaagttc
ctccctgaaagattcagcaagaagaacaaggacaacatagatccttacatatacacaccctttggaagtggacccagaaactgcattggcatgag
gtttgctctcatgaacatgaaacttgctctaatcagagtccttcagaacttctccttcaaaccttgtaaagaaacacagatccccctgaaattaagctta
ggaggacttcttcaaccagaaaaacccgttgttctaaaggttgagtcaagggatggcaccgtaagtggagccggatctggagcaacaaacttctc
actactcaaacaagcaggtgacgtggaggagaatcccgggcctatgatcaacatgggagactcccacgtggacaccagctccaccgtgtccga
ggcggtggccgaagaagtatctcttttcagcatgacggacatgattctgttttcgctcatcgtgggtctcctaacctactggttcctcttcagaaagaa
aaaagaagaagtccccgagttcaccaaaattcagacattgacctcctctgtcagagagagcagctttgtggaaaagatgaagaaaacggggagg
aacatcatcgtgttctacggctcccagacggggactgcagaggagtttgccaaccgcctgtccaaggacgcccaccgctacgggatgcgaggc
atgtcagcggaccctgaggagtatgacctggccgacctgagcagcctgccagagatcgacaacgccctggtggttttctgcatggccacctacg
gtgagggagaccccaccgacaatgcccaggacttctacgactggctgcaggagacagacgtggatctctctggggtcaagttcgcggtgtttgg
tcttgggaacaagacctacgagcacttcaatgccatgggcaagtacgtggacaagcggctggagcagctcggcgcccagcgcatctttgagct
ggggttgggcgacgacgatgggaacttggaggaggacttcatcacctggcgagagcagttctggccggccgtgtgtgaacactttggggtgga
agccactggcgaggagtccagcattcgccagtacgagcttgtggtccacaccgacatagatgcggccaaggtgtacatgggggagatgggcc
ggctgaagagctacgagaaccagaagccccccttgatgccaagaatccgttcctggctgcagtcaccaccaaccggaagctgaacca gggaa
ccgagcgccacctcatgcacctggaattggacatctcggactccaaaatcaggtatgaatctggggaccacgtggctgtgtacccagccaacga
ctctgctctcgtcaaccagctgggcaaaatcctgggtgccgacctggacgtcgtcatgtccctgaacaacctggatgaggagtccaacaagaag

cacccattcccgtgccctacgtcctaccgcacggccctcacctactacctggacatcaccaacccgccgcgtaccaacgtgctgtacgagctgg
cgcagtacgcctcggagccctcggagcaggagctgctgcgcaagatggcctcctcctccggcgagggcaaggagctgtacctgagctgggtg
gtggaggcccggaggcacatcctggccatcctgcaggactgcccgtccctgcggcccccatcgaccacctgtgtgagctgctgccgcgcctg
caggcccgctactactccatcgcctcatcctccaaggtccaccccaactctgtgcacatctgtgcggtggttgtggagtacgagaccaaggccgg
ccgcatcaacaagggcgtggccaccaactggctgcgggccaaggagcctgTcggggagaacggcggccgtgcgctggtgcccatgttcgtg
cgcaagtcccagttccgcctgcccttcaaggccaccacgcctgtcatcatggtgggccccggcaccggggtggcacccttcataggcttcatcc
aggagcgggcctggctgcgacagcagggcaaggaggtgggggagacgctgctgtactacggctgccgccgctcggatgaggactacctgta
ccgggaggagctggcgcagttccacagggacggtgcgctcacccagctcaacgtggccttctcccgggagcagtcccacaaggtctacgtcc
agcacctgctaaagcaagaccgagagcacctgtggaagttgatcgaaggcggtgcccacatctacgtctgtggggatgcacggaacatggcca
gggatgtgcagaacaccttctacgacatcgtggctgagctcggggccatggagcacgcgcaggcggtggactacatcaagaaactgatgacca
agggccgctactccctggacgtgtggagcggcagtggagagggcagaggaagtctgctaacatgcggtgacgtcgaggagaatcctggccc
aatgaccgagtacaagcccacggtgcgcctcgccacccgcgacgacgtccccagggccgtacgcaccctcgccgccgcgttcgccgactac
cccgccacgcgccacaccgtcgatccggaccgccacatcgagcgggtcaccgagctgcaagaactcttcctcacgcgcgtcgggctcgacat
cggcaaggtgtgggtcgcggacgacggcgccgcggtggcggtctggaccacgccggagagcgtcgaagcgggggcggtgttcgccgaga
tcggcccgcgcatggccgagttgagcggttcccggctggccgcgcagcaacagatggaaggcctcctggcgccgcaccggcccaaggagc
ccgcgtggttcctggccaccgtcggcgtctcgcccgaccaccagggcaagggtctgggcagcgccgtcgtgctccccggagtggaggcggc
cgagcgcgccggggtgcccgccttcctggagacctccgcgccccgcaacctccccttctacgagcggctcggcttcaccgtcaccgccgacgt
cgaggtgcccgaaggaccgcgcacctggtgcatgacccgcaagcccggtgcctgaGATCCACTAGTTCTAGAGCGGCC
GCGACTCTAGATCATAATCAGCCATACCACATTTGTAGAGGTTTTACTTGCTTTAAAAAAC
CTCCCACACCTCCCCCTGAACCTGAAACATAAAATGAATGCAATTGTTGTTGTTAACTTG
TTTATTGCAGCTTATAATGGTTACAAATAAAGCAATAGCATCACAAATTTCACAAATAAAG
CATTTTTTTCACTGCATTCTAGTTGTGGTTTGTCCAAACTCATCAATGTATCTTAcacttccgaatt
ggagccgcttcaactggccctgggcttagccactctgtgctgaccactctgccccaggcctccttaccattccccttcgacctactctccaaacacg
tacgcgtacgatgcAGGCGTAAATTGTAAGCGTTAATATTTTGTTAAAATTCGCGTTAAATTTTTGT
TAAATCAGCTCATTTTTTAACCAATAGGCCGAAATCGGCAAAATCCCTTATAAATCAAAAG
AATAGACCGAGATAGGGTTGAGTGTTGTTCCAGTTTGGAACAAGAGTCCACTATTAAAGA
ACGTGGACTCCAACGTCAAAGGGCGAAAAACCGTCTATCAGGGCGATGGCCCACTACGT
GAACCATCACCCTAATCAAGTTTTTTGGGGTCGAGGTGCCGTAAAGCACTAAATCGGAAC
CCTAAAGGGAGCCCCCGATTTAGAGCTTGACGGGGAAAGCCGGCGAACGTGGCGAGAA
AGGAAGGGAAGAAAGCGAAAGGAGCGGGCGCTAGGGCGCTGGCAAGTGTAGCGGTCA
CGCTGCGCGTAACCACCACACCCGCCGCGCTTAATGCGCCGCTACAGGGCGCGTCAGGT
GGCACTTTTCGGGGAAATGTGCGCGGAACCCCTATTTGTTTATTTTTCTAAATACATTCAA
ATATGTATCCGCTCATGAGACAATAACCCTGATAAATGCTTCAATAATATTGAAAAAGGAA
GAGTCCTGAGGCGGAAAGAACCAGCTGTGGAATGTGTGTCAGTTAGGGTGTGGAAAGT
CCCCAGGCTCCCCAGCAGGCAGAAGTATGCAAAGCATGCATCTCAATTAGTCAGCAACC
AGGTGTGGAAAGTCCCCAGGCTCCCCAGCAGGCAGAAGTATGCAAAGCATGCATCTCAA

TTAGTCAGCAACCATAGTCCCGCCCCTAACTCCGCCCATCCCGCCCCTAACTCCGCCCAG
TTCCGCCCATTCTCCGCCCCATGGCTGACTAATTTTTTTTATTTATGCAGAGGCCGAGGCC
GCCTCGGCCTCTGAGCTATTCCAGAAGTAGTGAGGAGGCTTTTTTGGAGGCCTAGGCTTT
TGCAAAGATCATCAAGAGACAGGATGAGGATCGTTTCGCATGATTGAACAAGATGGATTG
CACGCAGGTTCTCCGGCCGCTTGGGTGGAGAGGCTATTCGGCTATGACTGGGCACAACA
GACAATCGGCTGCTCTGATGCCGCCGTGTTCCGGCTGTCAGCGCAGGGGCGCCCGGTTC
TTTTTGTCAAGACCGACCTGTCCGGTGCCCTGAATGAACTGCAAGACGAGGCAGCGCGG
CTATCGTGGCTGGCCACGACGGGCGTTCCTTGCGCAGCTGTGCTCGACGTTGTCACTGAA
GCGGGAAGGGACTGGCTGCTATTGGGCGAAGTGCCGGGGCAGGATCTCCTGTCATCTCA
CCTTGCTCCTGCCGAGAAAGTATCCATCATGGCTGATGCAATGCGGCGGCTGCATACGCT
TGATCCGGCTACCTGCCCATTCGACCACCAAGCGAAACATCGCATCGAGCGAGCACGTA
CTCGGATGGAAGCCGGTCTTGTCGATCAGGATGATCTGGACGAAGAGCATCAGGGGCTC
GCGCCAGCCGAACTGTTCGCCAGGCTCAAGGCGAGCATGCCCGACGGCGAGGATCTCGT
CGTGACCCATGGCGATGCCTGCTTGCCGAATATCATGGTGGAAAATGGCCGCTTTTCTGG
ATTCATCGACTGTGGCCGGCTGGGTGTGGCGGACCGCTATCAGGACATAGCGTTGGCTAC
CCGTGATATTGCTGAAGAGCTTGGCGGCGAATGGGCTGACCGCTTCCTCGTGCTTTACGG
TATCGCCGCTCCCGATTCGCAGCGCATCGCCTTCTATCGCCTTCTTGACGAGTTCTTCTGA
GCGGGACTCTGGGGTTCGAAATGACCGACCAAGCGACGCCCAACCTGCCATCACGAGAT
TTCGATTCCACCGCCGCCTTCTATGAAAGGTTGGGCTTCGGAATCGTTTTCCGGGACGCC
GGCTGGATGATCCTCCAGCGCGGGGATCTCATGCTGGAGTTCTTCGCCCACCCTAGGGGG
AGGCTAACTGAAACACGGAAGGAGACAATACCGGAAGGAACCCGCGCTATGACGGCAA
TAAAAAGACAGAATAAAACGCACGGTGTTGGGTCGTTTGTTCATAAACGCGGGGTTCGG
TCCCAGGGCTGGCACTCTGTCGATACCCCACCGAGACCCCATTGGGGCCAATACGCCCGC
GTTTCTTCCTTTTCCCCACCCCACCCCCCAAGTTCGGGTGAAGGCCCAGGGCTCGCAGCC
AACGTCGGGGCGGCAGGCCCTGCCATAGCCTCAGGTTACTCATATATACTTTAGATTGATT
TAAAACTTCATTTTTAATTTAAAAGGATCTAGGTGAAGATCCTTTTTGATAATCTCATGACC
AAAATCCCTTAACGTGAGTTTTCGTTCCACTGAGCGTCAGACCCCGTAGAAAAGATCAA
AGGATCTTCTTGAGATCCTTTTTTTCTGCGCGTAATCTGCTGCTTGCAAACAAAAAAACC
ACCGCTACCAGCGGTGGTTTGTTTGCCGGATCAAGAGCTACCAACTCTTTTTCCGAAGGT
AACTGGCTTCAGCAGAGCGCAGATACCAAATACTGTTCTTCTAGTGTAGCCGTAGTTAGG
CCACCACTTCAAGAACTCTGTAGCACCGCCTACATACCTCGCTCTGCTAATCCTGTTACCA
GTGGCTGCTGCCAGTGGCGATAAGTCGTGTCTTACCGGGTTGGACTCAAGACGATAGTTA
CCGGATAAGGCGCAGCGGTCGGGCTGAACGGGGGGTTCGTGCACACAGCCCAGCTTGG
AGCGAACGACCTACACCGAACTGAGATACCTACAGCGTGAGCTATGAGAAAGCGCCACG
CTTCCCGAAGGGAGAAAGGCGGACAGGTATCCGGTAAGCGGCAGGGTCGGAACAGGAG
AGCGCACGAGGGAGCTTCCAGGGGGAAACGCCTGGTATCTTTATAGTCCTGTCGGGTTTC

GCCACCTCTGACTTGAGCGTCGATTTTTGTGATGCTCGTCAGGGGGGCGGAGCCTATGGA
AAAACGCCAGCAACGCGGCCTTTTTACGGTTCCTGGCCTTTTGCTGGCCTTTTGCTCACA
TGTTCTTTCCTGCGTTATCCCCTGATTCTGTGGATAACCGTATTACCGCCATGCAT

Donor Plasmid (PITCh CCR5 pCAG-UGT1A1-E2A-NeoR-pA)

Full sequences of PITCh CCR5 pCAG-UGT1A1-E2A-NeoR-pA donor plasmid is shown below.

PITCh gRNA target site, CCR5 homology arm, UGT1A1, NeoR

gcatcgtacgcgtacgtgtttggtagtcttcttccaaaagcacattgccaaacgcttctgcaaatgctgttctattttccagcaagaggctcccgagc
gagcaagctcagtttacacccgatcTAGTTATTAATAGTAATCAATTACGGGGTCATTAGTTCATAGCCCA
TATATGGAGTTCCGCGTTACATAACTTACGGTAAATGGCCCGCCTGGCTGACCGCCCAAC
GACCCCCGCCCATTGACGTCAATAATGACGTATGTTCCCATAGTAACGCCAATAGGGACTT
TCCATTGACGTCAATGGGTGGAGTATTTACGGTAAACTGCCCACTTGGCAGTACATCAAG
TGTATCATATGCCAAGTACGCCCCCTATTGACGTCAATGACGGTAAATGGCCCGCCTGGCA
TTATGCCCAGTACATGACCTTATGGGACTTTCCTACTTGGCAGTACATCTACGTATTAGTCA
TCGCTATTACCATGGTCGAGGTGAGCCCCACGTTCTGCTTCACTCTCCCCATCTCCCCCCC
CTCCCCACCCCCAATTTTGTATTTATTTATTTTTTAATTATTTTGTGCAGCGATGGGGGCGG
GGGGGGGGGGGGGGCGCGCGCCAGGCGGGGCGGGGCGGGGCGAGGGGCGGGGCGGG
GCGAGGCGGAGAGGTGCGGCGGCAGCCAATCAGAGCGGCGCGCTCCGAAAGTTTCCTT
TTATGGCGAGGCGGCGGCGGCGGCGGCCCTATAAAAAGCGAAGCGCGCGGCGGGCGGG
AGTCGCTGCGCGCTGCCTTCGCCCCGTGCCCCGCTCCGCCGCCGCCTCGCGCCGCCCGC
CCCGGCTCTGACTGACCGCGTTACTCCCACAGGTGAGCGGGCGGGACGGCCCTTCTCCT
CCGGGCTGTAATTAGCGCTTGGTTTAATGACGGCTTGTTTCTTTTCTGTGGCTGCGTGAAA
GCCTTGAGGGGCTCCGGGAGGGCCCTTTGTGCGGGGGGAGCGGCTCGGGGGGTGCGTG
CGTGTGTGTGTGCGTGGGGAGCGCCGCGTGCGGCTCCGCGCTGCCCGGCGGCTGTGAGC
GCTGCGGGCGCGGCGCGGGGCTTTGTGCGCTCCGCAGTGTGCGCGAGGGGAGCGCGGC
CGGGGGCGGTGCCCCGCGGTGCGGGGGGGGCTGCGAGGGGAACAAAGGCTGCGTGCG
GGGTGTGTGCGTGGGGGGGTGAGCAGGGGGTGTGGGCGCGTCGGTCGGGCTGCAACCC
CCCCTGCACCCCCCTCCCCGAGTTGCTGAGCACGGCCCGGCTTCGGGTGCGGGGCTCCG
TACGGGGCGTGGCGCGGGGCTCGCCGTGCCGGGCGGGGGGTGGCGGCAGGTGGGGGTG
CCGGGCGGGGCGGGGCCGCCTCGGGCCGGGGAGGGCTCGGGGGAGGGGCGCGGCGGC
CCCCGGAGCGCCGGCGGCTGTCGAGGCGCGGCGAGCCGCAGCCATTGCCTTTTATGGTA
ATCGTGCGAGAGGGCGCAGGGACTTCCTTTGTCCCAAATCTGTGCGGAGCCGAAATCTG
GGAGGCGCCGCCGCACCCCCTCTAGCGGGCGCGGGGCGAAGCGGTGCGGCGCCGGCAG
GAAGGAAATGGGCGGGGAGGGCCTTCGTGCGTCGCCGCGCCGCCGTCCCCTTCTCCCTC
TCCAGCCTCGGGGCTGTCCGCGGGGGGACGGCTGCCTTCGGGGGGGACGGGGCAGGGC

GGGGTTCGGCTTCTGGCGTGTGACCGGCGGCTCTAGAGCCTCTGCTAACCATGTTCATGC
CTTCTTCTTTTTCCTACAGCTCCTGGGCAACGTGCTGGTTATTGTGCTGTCTCATCATTTTG
GCAAAGAATTAACCCTCACTAAAGGGGTACCGGGCCCCCCCTCGAGGTCGACGGTATCG
ATAAGCTTGATATCGAATTgccgccatggctgtggagtcccagggcggacgcccacttgtcctgggcctgctgctgtgtgtgctg
ggcccagtggtgtcccatgctgggaagatactgttgatcccagtggatggcagccactggctgagcatgcttggggccatccagcagctgcagc
agaggggacatgaaatagttgtcctagcacctgacgcctcgttgtacatcagagacggagcattttacaccttgaagacgtaccctgtgccattcc
aaagggaggatgtgaaagagtcttttgttagtctcgggcataatgtttttgagaatgattctttcctgcagcgtgtgatcaaaacatacaagaaaataa
aaaaggactctgctatgcttttgtctggctgttcccacttactgcacaacaaggagctcatggcctccctggcagaaagcagctttgatgtcatgctg
acggaccctttccttccttgcagccccatcgtggcccagtacctgtctctgcccactgtattcttcttgcatgcactgccatgcagcctggaatttgag
gctacccagtgccccaacccattctcctacgtgcccaggcctctctcctctcattcagatcacatgaccttcctgcagcgggtgaagaacatgctca
ttgccttttcacagaactttctgtgcgacgtggtttattccccgtatgcaacccttgcctcagaattccttcagagagaggtgactgtccaggacctatt
gagctctgcatctgtctggctgtttagaagtgactttgtgaaggattaccctaggcccatcatgcccaatatggtttttgttggtggaatcaactgcctt
caccaaaatccactatcccaggaatttgaagcctacattaatgcttctggagaacatggaattgtggttttctctttgggatcaatggtctcagaaattc
cagagaagaaagctatggcaattgctgatgctttgggcaaaatccctcagacagtcctgtggcggtacactggaacccgaccatcgaatcttgcg
aacaacacgatacttgttaagtggctaccccaaaacgatctgcttggtcacccgatgacccgtgcctttatcacccatgctggttcccatggtgtttat
gaaagcatatgcaatggcgttcccatggtgatgatgcccttgtttggtgatcagatggacaatgcaaagcgcatggagactaagggagctggagt
gaccctgaatgttctggaaatgacttctgaagatttagaaaatgctctaaaagcagtcatcaatgacaaaagttacaaggagaacatcatgcgcctc
tccagccttcacaaggaccgcccggtggagccgctggacctggccgtgttctgggtggagtttgtgatgaggcacaagggcgcgccacacctg
cgccccgcagcccacgacctcacctggtaccagtaccattccttggacgtgattggtttcctcttggccgtcgtgctgacagtggccttcatcacctt
taaatgttgtgcttatggctaccggaaatgcttggggaaaaaagggcgagttaagaaagcccacaaatccaagacccatGGAAGCGGA
CAGTGTACTAATTATGCTCTCTTGAAATTGGCTGGAGATGTTGAGAGCAACCCTGGACCT
ATGATTGAACAAGATGGATTGCACGCAGGTTCTCCGGCCGCTTGGGTGGAGAGGCTATTC
GGCTATGACTGGGCACAACAGACAATCGGCTGCTCTGATGCCGCCGTGTTCCGGCTGTCA
GCGCAGGGGCGCCCGGTTCTTTTTGTCAAGACCGACCTGTCCGGTGCCCTGAATGAACT
GCAAGACGAGGCAGCGCGGCTATCGTGGCTGGCCACGACGGGCGTTCCTTGCGCAGCTG
TGCTCGACGTTGTCACTGAAGCGGGAAGGGACTGGCTGCTATTGGGCGAAGTGCCGGGG
CAGGATCTCCTGTCATCTCACCTTGCTCCTGCCGAGAAAGTATCCATCATGGCTGATGCAA
TGCGGCGGCTGCATACGCTTGATCCGGCTACCTGCCCATTCGACCACCAAGCGAAACATC
GCATCGAGCGAGCACGTACTCGGATGGAAGCCGGTCTTGTCGATCAGGATGATCTGGAC
GAAGAGCATCAGGGGCTCGCGCCAGCCGAACTGTTCGCCAGGCTCAAGGCGAGCATGC
CCGACGGCGAGGATCTCGTCGTGACCCATGGCGATGCCTGCTTGCCGAATATCATGGTGG
AAAATGGCCGCTTTTCTGGATTCATCGACTGTGGCCGGCTGGGTGTGGCGGACCGCTATC
AGGACATAGCGTTGGCTACCCGTGATATTGCTGAAGAGCTTGGCGGCGAATGGGCTGACC
GCTTCCTCGTGCTTTACGGTATCGCCGCTCCCGATTCGCAGCGCATCGCCTTCTATCGCCT
TCTTGACGAGTTCTTCTGAGGATCCACTAGTTCTAGAGCGGCCGCGACTCTAGATCATAAT
CAGCCATACCACATTTGTAGAGGTTTTACTTGCTTTAAAAAACCTCCCACACCTCCCCCTG

AACCTGAAACATAAAATGAATGCAATTGTTGTTGTTAACTTGTTTATTGCAGCTTATAATG
GTTACAAATAAAGCAATAGCATCACAAATTTCACAAATAAAGCATTTTTTTCACTGCATTC
TAGTTGTGGTTTGTCCAAACTCATCAATGTATCTTActggggagcaggaaatatctgtgggcttgtgacacggac
tcaagtgggctggtgacccagtcagagttgtgcacatggcttagttttcatacacagcctgccaaacacgtacgcgtacgatgcAGGCGTA
AATTGTAAGCGTTAATATTTTGTTAAAATTCGCGTTAAATTTTTGTTAAATCAGCTCATTTT
TTAACCAATAGGCCGAAATCGGCAAAATCCCTTATAAATCAAAAGAATAGACCGAGATAG
GGTTGAGTGTTGTTCCAGTTTGGAACAAGAGTCCACTATTAAAGAACGTGGACTCCAAC
GTCAAAGGGCGAAAAACCGTCTATCAGGGCGATGGCCCACTACGTGAACCATCACCCTA
ATCAAGTTTTTTGGGGTCGAGGTGCCGTAAAGCACTAAATCGGAACCCTAAAGGGAGCC
CCCGATTTAGAGCTTGACGGGGAAAGCCGGCGAACGTGGCGAGAAAGGAAGGGAAGAA
AGCGAAAGGAGCGGGCGCTAGGGCGCTGGCAAGTGTAGCGGTCACGCTGCGCGTAACC
ACCACACCCGCCGCGCTTAATGCGCCGCTACAGGGCGCGTCAGGTGGCACTTTTCGGGG
AAATGTGCGCGGAACCCCTATTTGTTTATTTTTCTAAATACATTCAAATATGTATCCGCTCA
TGAGACAATAACCCTGATAAATGCTTCAATAATATTGAAAAAGGAAGAGTCCTGAGGCGG
AAAGAACCAGCTGTGGAATGTGTGTCAGTTAGGGTGTGGAAAGTCCCCAGGCTCCCCAG
CAGGCAGAAGTATGCAAAGCATGCATCTCAATTAGTCAGCAACCAGGTGTGGAAAGTCC
CCAGGCTCCCCAGCAGGCAGAAGTATGCAAAGCATGCATCTCAATTAGTCAGCAACCATA
GTCCCGCCCCTAACTCCGCCCATCCCGCCCCTAACTCCGCCCAGTTCCGCCCATTCTCCG
CCCCATGGCTGACTAATTTTTTTTATTTATGCAGAGGCCGAGGCCGCCTCGGCCTCTGAGC
TATTCCAGAAGTAGTGAGGAGGCTTTTTTGGAGGCCTAGGCTTTTGCAAAGATCATCAAG
AGACAGGATGAGGATCGTTTCGCATGATTGAACAAGATGGATTGCACGCAGGTTCTCCG
GCCGCTTGGGTGGAGAGGCTATTCGGCTATGACTGGGCACAACAGACAATCGGCTGCTC
TGATGCCGCCGTGTTCCGGCTGTCAGCGCAGGGGCGCCCGGTTCTTTTTGTCAAGACCG
ACCTGTCCGGTGCCCTGAATGAACTGCAAGACGAGGCAGCGCGGCTATCGTGGCTGGCC
ACGACGGGCGTTCCTTGCGCAGCTGTGCTCGACGTTGTCACTGAAGCGGGAAGGGACTG
GCTGCTATTGGGCGAAGTGCCGGGGCAGGATCTCCTGTCATCTCACCTTGCTCCTGCCGA
GAAAGTATCCATCATGGCTGATGCAATGCGGCGGCTGCATACGCTTGATCCGGCTACCTG
CCCATTCGACCACCAAGCGAAACATCGCATCGAGCGAGCACGTACTCGGATGGAAGCCG
GTCTTGTCGATCAGGATGATCTGGACGAAGAGCATCAGGGGCTCGCGCCAGCCGAACTG
TTCGCCAGGCTCAAGGCGAGCATGCCCGACGGCGAGGATCTCGTCGTGACCCATGGCGA
TGCCTGCTTGCCGAATATCATGGTGGAAAATGGCCGCTTTTCTGGATTCATCGACTGTGGC
CGGCTGGGTGTGGCGGACCGCTATCAGGACATAGCGTTGGCTACCCGTGATATTGCTGAA
GAGCTTGGCGGCGAATGGGCTGACCGCTTCCTCGTGCTTTACGGTATCGCCGCTCCCGAT
TCGCAGCGCATCGCCTTCTATCGCCTTCTTGACGAGTTCTTCTGAGCGGGACTCTGGGGT
TCGAAATGACCGACCAAGCGACGCCCAACCTGCCATCACGAGATTTCGATTCCACCGCC
GCCTTCTATGAAAGGTTGGGCTTCGGAATCGTTTTCCGGGACGCCGGCTGGATGATCCTC

CAGCGCGGGGATCTCATGCTGGAGTTCTTCGCCCACCCTAGGGGGAGGCTAACTGAAAC
ACGGAAGGAGACAATACCGGAAGGAACCCGCGCTATGACGGCAATAAAAAGACAGAAT
AAAACGCACGGTGTTGGGTCGTTTGTTCATAAACGCGGGGTTCGGTCCCAGGGCTGGCA
CTCTGTCGATACCCCACCGAGACCCCATTGGGGCCAATACGCCCGCGTTTCTTCCTTTTCC
CCACCCCACCCCCCAAGTTCGGGTGAAGGCCCAGGGCTCGCAGCCAACGTCGGGGCGG
CAGGCCCTGCCATAGCCTCAGGTTACTCATATATACTTTAGATTGATTTAAAACTTCATTTT
TAATTTAAAAGGATCTAGGTGAAGATCCTTTTTGATAATCTCATGACCAAAATCCCTTAAC
GTGAGTTTTCGTTCCACTGAGCGTCAGACCCCGTAGAAAAGATCAAAGGATCTTCTTGA
GATCCTTTTTTTCTGCGCGTAATCTGCTGCTTGCAAACAAAAAAACCACCGCTACCAGCG
GTGGTTTGTTTGCCGGATCAAGAGCTACCAACTCTTTTTCCGAAGGTAACTGGCTTCAGC
AGAGCGCAGATACCAAATACTGTTCTTCTAGTGTAGCCGTAGTTAGGCCACCACTTCAAG
AACTCTGTAGCACCGCCTACATACCTCGCTCTGCTAATCCTGTTACCAGTGGCTGCTGCCA
GTGGCGATAAGTCGTGTCTTACCGGGTTGGACTCAAGACGATAGTTACCGGATAAGGCGC
AGCGGTCGGGCTGAACGGGGGGTTCGTGCACACAGCCCAGCTTGGAGCGAACGACCTA
CACCGAACTGAGATACCTACAGCGTGAGCTATGAGAAAGCGCCACGCTTCCCGAAGGGA
GAAAGGCGGACAGGTATCCGGTAAGCGGCAGGGTCGGAACAGGAGAGCGCACGAGGG
AGCTTCCAGGGGGAAACGCCTGGTATCTTTATAGTCCTGTCGGGTTTCGCCACCTCTGAC
TTGAGCGTCGATTTTTGTGATGCTCGTCAGGGGGGCGGAGCCTATGGAAAAACGCCAGC
AACGCGGCCTTTTTACGGTTCCTGGCCTTTTGCTGGCCTTTTGCTCACATGTTCTTTCCTG
CGTTATCCCCTGATTCTGTGGATAACCGTATTACCGCCATGCAT

Donor Plasmid (PITCh CYP3A7 pCAG-CYP1A2-P2A-CYP2C19-T2A-BleoR-pA)

Full sequences of PITCh CYP3A7 pCAG-CYP1A2-P2A-CYP2C19-T2A-BleoR-pA donor plasmid is shown below.

PITCh gRNA target site, CYP3A7 homology arm, CYP1A2, CYP2C19, BleoR

gcatcgtacgcgtacgtgtttggcattattgtcagtaatttttttttactttgatgctatactttctacttttgctttatttaatgcttctcaatatgctcgtttaact
gttgcagatccccc TAGTTATTAATAGTAATCAATTACGGGGTCATTAGTTCATAGCCCATATATGG
AGTTCCGCGTTACATAACTTACGGTAAATGGCCCGCCTGGCTGACCGCCCAACGACCCCC
GCCCATTGACGTCAATAATGACGTATGTTCCCATAGTAACGCCAATAGGGACTTTCCATTG
ACGTCAATGGGTGGAGTATTTACGGTAAACTGCCCACTTGGCAGTACATCAAGTGTATCA
TATGCCAAGTACGCCCCCTATTGACGTCAATGACGGTAAATGGCCCGCCTGGCATTATGCC
CAGTACATGACCTTATGGGACTTTCCTACTTGGCAGTACATCTACGTATTAGTCATCGCTAT
TACCATGGTCGAGGTGAGCCCCACGTTCTGCTTCACTCTCCCCATCTCCCCCCCCTCCCC
ACCCCCAATTTTGTATTTATTTATTTTTTAATTATTTTGTGCAGCGATGGGGGCGGGGGGGG
GGGGGGGGCGCGCGCCAGGCGGGGCGGGGCGGGGCGAGGGGCGGGGCGGGGCGAGGC
GGAGAGGTGCGGCGGCAGCCAATCAGAGCGGCGCGCTCCGAAAGTTTCCTTTTATGGCG

AGGCGGCGGCGGCGGCGGCCCTATAAAAAGCGAAGCGCGCGGCGGGCGGGAGTCGCTG
CGCGCTGCCTTCGCCCCGTGCCCCGCTCCGCCGCCGCCTCGCGCCGCCCGCCCCGGCTCT
GACTGACCGCGTTACTCCCACAGGTGAGCGGGCGGGACGGCCCTTCTCCTCCGGGCTGT
AATTAGCGCTTGGTTTAATGACGGCTTGTTTCTTTTCTGTGGCTGCGTGAAAGCCTTGAG
GGGCTCCGGGAGGGCCCTTTGTGCGGGGGGAGCGGCTCGGGGGGTGCGTGCGTGTGTG
TGTGCGTGGGGAGCGCCGCGTGCGGCTCCGCGCTGCCCGGCGGCTGTGAGCGCTGCGG
GCGCGGCGCGGGGCTTTGTGCGCTCCGCAGTGTGCGCGAGGGGAGCGCGGCCGGGGGC
GGTGCCCCGCGGTGCGGGGGGGGCTGCGAGGGGAACAAAGGCTGCGTGCGGGGTGTGT
GCGTGGGGGGGTGAGCAGGGGGTGTGGGCGCGTCGGTCGGGCTGCAACCCCCCCTGCA
CCCCCCTCCCCGAGTTGCTGAGCACGGCCCGGCTTCGGGTGCGGGGCTCCGTACGGGGC
GTGGCGCGGGGCTCGCCGTGCCGGGCGGGGGGTGGCGGCAGGTGGGGGTGCCGGGCGG
GGCGGGGCCGCCTCGGGCCGGGGAGGGCTCGGGGGAGGGGCGCGGCGGCCCCCGGAG
CGCCGGCGGCTGTCGAGGCGCGGCGAGCCGCAGCCATTGCCTTTTATGGTAATCGTGCG
AGAGGGCGCAGGGACTTCCTTTGTCCCAAATCTGTGCGGAGCCGAAATCTGGGAGGCGC
CGCCGCACCCCCTCTAGCGGGCGCGGGGCGAAGCGGTGCGGCGCCGGCAGGAAGGAAA
TGGGCGGGGAGGGCCTTCGTGCGTCGCCGCGCCGCCGTCCCCTTCTCCCTCTCCAGCCT
CGGGGCTGTCCGCGGGGGGACGGCTGCCTTCGGGGGGGACGGGGCAGGGCGGGGTTCG
GCTTCTGGCGTGTGACCGGCGGCTCTAGAGCCTCTGCTAACCATGTTCATGCCTTCTTCTT
TTTCCTACAGCTCCTGGGCAACGTGCTGGTTATTGTGCTGTCTCATCATTTTGGCAAAGAA
TTAACCCTCACTAAAGGGTACCGGGCCCCCCCTCGAGGTCGACGGTATCGATAAGCTTG
ATATCGAATTgccgccatggcattgtcccagtctgttcccttctcggccacagagcttctcctggcctctgccatcttctgcctggtattctgg
gtgctcaagggtttgaggcctcgggtccccaaaggcctgaaaagtccaccagagccatggggctggcccttgctcgggcatgtgctgaccctg
gggaagaacccgcacctggcactgtcaaggatgagccagcgctacggggacgtcctgcagatccgcattggctccacgcccgtgctggtgct
gagccgcctggacaccatccggcaggccctggtgcggcagggcgacgatttcaagggccggcctgacctctacacctccaccctcatcactga
tggccagagcttgaccttcagcacagactctggaccggtgtgggctgcccgccggcgcctggcccagaatgccctcaacaccttctccatcgcc
tctgacccagcttcctcatcctcctgctacctggaggagcatgtgagcaaggaggctaaggccctgatcagcaggttgcaggagctgatggcag
ggcctgggcacttcgacccttacaatcaggtggtggtgtcagtggccaacgtcattggtgccatgtgcttcggacagcacttccctgagagtagcg
atgagatgctcagcctcgtgaagaacactcatgagttcgtggagactgcctcctccgggaacccccctggacttcttccccatccttcgctacctgcc
taaccctgccctgcagaggttcaaggccttcaaccagaggttcctgtggttcctgcagaaaacagtccaggagcactatcaggactttgacaaga
acagtgtccgggacatcacgggtgccctgttcaagcacagcaagaaggggcctagagccagcggcaacctcatcccacaggagaagattgtc
aaccttgtcaatgacatctttggagcaggatttgacacagtcaccacagccatctcctggagcctcatgtaccttgtgaccaagcctgagatacaga
ggaagatccagaaggagctggacactgtgattggcagggagcggcggccccggctctctgacagaccccagctgccctacttggaggccttc
atcctggagaccttccgacactcctccttcttgcccttcaccatcccccacagcacaacaagggacacaacgctgaatggcttctacatccccaag
aaatgctgtgtcttcgtaaaccagtggcaggtcaaccatgacccagagctgtgggaggacccctctgagttccggcctgagcggttcctcaccgc
cgatggcactgccattaacaagcccttgagtgagaagatgatgctgtttggcatgggcaagcgccggtgtatcggggaagtcctggccaagtgg
gagatcttcctcttcctggccatcctgctacagcaactggagttcagcgtgccgccgggcgtgaaagtcgacctgacccccatctacgggctgac

catgaagcacgcccgctgtgaacatgtccaggcgcggctgcgcttctccatcaatggatctggagcaacaaacttctcactactcaaacaagcag
gtgacgtggaggagaatcccgggcctatggatccttttgtggtccttgtgctctgtctctcatgtttgcttctcctttcaatctggagacagagctctgg
gagaggaaaactccctcctggcccTactcctctcccagtgattggaaatatcctacagatagatattaaggatgtcagcaaatccttaaccaatctc
tcaaaaatctatggccctgtgttcactctgtattttggcctggaacgcatggtggtgctgcatggatatgaagtggtgaaggaagccctgattgatctt
ggagaggagttttctggaagaggccatttcccactggctgaaagagctaacagaggatttggaatcgttttcagcaatggaaagagatggaagga
gatccggcgtttctccctcatgacgctgcggaattttgggatggggaagaggagcattgaggaccgtgttcaagaggaagcccgctgccttgtgg
aggagttgagaaaaaccaaggcttcaccctgtgatcccactttcatcctgggctgtgctccctgcaatgtgatctgctccattattttccagaaacgtt
tcgattataaagatcagcaatttcttaacttgatggaaaaattgaatgaaaacatcaggattgtaagcaccccctggatccagatatgcaataattttcc
cactatcattgattatttcccgggaacccataacaaattacttaaaaaccttgcttttatggaaagtgatattttggagaaagtaaaagaacaccaaga
atcgatggacatcaacaaccctcgggactttattgattgcttcctgatcaaaatggagaaggaaaagcaaaaccaacagtctgaattcactattgaa
aacttggtaatcactgcagctgacttacttggagctgggacagagacaacaagcacaaccctgagatatgctctccttctcctgctgaagcaccca
gaggtcacagctaaagtccaggaagagattgaacgtgtcGttggcagaaaccggagcccctgcatgcaggacaggggccacatgccctacac
agatgctgtggtgcacgaggtccagagatacatcgacctcatccccaccagcctgccccatgcagtgacctgtgacgttaaattcagaaactacct
cattcccaagggcacaaccatattaacttccctcacttctgtgctacatgacaacaaagaatttcccaacccagagatgtttgaccctcgtcactttct
ggatgaaggtggaaattttaagaaaagtaactacttcatgcctttctcagcaggaaaacggatttgtgtgggagagggcctggcccgcatggagct
gtttttattcctgaccttcattttacagaactttaacctgaaatctctgattgacccaaaggaccttgacacaactcctgttgtcaatggatttgcttctgtc
ccgcccttctatcagctgtgcttcattcctgtcggcagtggagagggcagaggaagtctgctaacatgcggtgacgtcgaggagaatcctggccc
aATGGCCAAGTTGACCAGTGCCGTTCCGGTGCTCACCGCGCGCGACGTCGCCGGAGCGG
TCGAGTTCTGGACCGACCGGCTCGGGTTCTCCCGGGACTTCGTGGAGGACGACTTCGCC
GGTGTGGTCCGGGACGACGTGACCCTGTTCATCAGCGCGGTCCAGGACCAGGTGGTGCC
GGACAACACCCTGGCCTGGGTGTGGGTGCGCGGCCTGGACGAGCTGTACGCCGAGTGG
TCGGAGGTCGTGTCCACGAACTTCCGGGACGCCTCCGGGCCGGCCATGACCGAGATCGG
CGAGCAGCCGTGGGGGCGGGAGTTCGCCCTGCGCGACCCGGCCGGCAACTGCGTGCAC
TTCGTGGCCGAGGAGCAGGACTGAGATCCACTAGTTCTAGAGCGGCCGCGACTCTAGAT
CATAATCAGCCATACCACATTTGTAGAGGTTTTACTTGCTTTAAAAAACCTCCCACACCTC
CCCCTGAACCTGAAACATAAAATGAATGCAATTGTTGTTGTTAACTTGTTTATTGCAGCTT
ATAATGGTTACAAATAAAGCAATAGCATCACAAATTTCACAAATAAAGCATTTTTTTCACT
GCATTCTAGTTGTGGTTTGTCCAAACTCATCAATGTATCTTAaattacgctttggaggacttcttctaacagaa
aaacccattgttctaaaggctgagtcaagggatgagaccgtaagtggagcctgatttccctaaggactccaaacacgtacgcgtacgatgcAG
GCGTAAATTGTAAGCGTTAATATTTTGTTAAAATTCGCGTTAAATTTTTGTTAAATCAGCTC
ATTTTTTAACCAATAGGCCGAAATCGGCAAAATCCCTTATAAATCAAAAGAATAGACCGA
GATAGGGTTGAGTGTTGTTCCAGTTTGGAACAAGAGTCCACTATTAAAGAACGTGGACTC
CAACGTCAAAGGGCGAAAAACCGTCTATCAGGGCGATGGCCCACTACGTGAACCATCAC
CCTAATCAAGTTTTTTGGGGTCGAGGTGCCGTAAAGCACTAAATCGGAACCCTAAAGGG
AGCCCCCGATTTAGAGCTTGACGGGGAAAGCCGGCGAACGTGGCGAGAAAGGAAGGGA
AGAAAGCGAAAGGAGCGGGCGCTAGGGCGCTGGCAAGTGTAGCGGTCACGCTGCGCGT

AACCACCACACCCGCCGCGCTTAATGCGCCGCTACAGGGCGCGTCAGGTGGCACTTTTC
GGGGAAATGTGCGCGGAACCCCTATTTGTTTATTTTTCTAAATACATTCAAATATGTATCCG
CTCATGAGACAATAACCCTGATAAATGCTTCAATAATATTGAAAAAGGAAGAGTCCTGAG
GCGGAAAGAACCAGCTGTGGAATGTGTGTCAGTTAGGGTGTGGAAAGTCCCCAGGCTCC
CCAGCAGGCAGAAGTATGCAAAGCATGCATCTCAATTAGTCAGCAACCAGGTGTGGAAA
GTCCCCAGGCTCCCCAGCAGGCAGAAGTATGCAAAGCATGCATCTCAATTAGTCAGCAA
CCATAGTCCCGCCCCTAACTCCGCCCATCCCGCCCCTAACTCCGCCCAGTTCCGCCCATTC
TCCGCCCCATGGCTGACTAATTTTTTTTATTTATGCAGAGGCCGAGGCCGCCTCGGCCTCT
GAGCTATTCCAGAAGTAGTGAGGAGGCTTTTTTGGAGGCCTAGGCTTTTGCAAAGATCAT
CAAGAGACAGGATGAGGATCGTTTCGCATGATTGAACAAGATGGATTGCACGCAGGTTC
TCCGGCCGCTTGGGTGGAGAGGCTATTCGGCTATGACTGGGCACAACAGACAATCGGCT
GCTCTGATGCCGCCGTGTTCCGGCTGTCAGCGCAGGGGCGCCCGGTTCTTTTTGTCAAGA
CCGACCTGTCCGGTGCCCTGAATGAACTGCAAGACGAGGCAGCGCGGCTATCGTGGCTG
GCCACGACGGGCGTTCCTTGCGCAGCTGTGCTCGACGTTGTCACTGAAGCGGGAAGGG
ACTGGCTGCTATTGGGCGAAGTGCCGGGGCAGGATCTCCTGTCATCTCACCTTGCTCCTG
CCGAGAAAGTATCCATCATGGCTGATGCAATGCGGCGGCTGCATACGCTTGATCCGGCTA
CCTGCCCATTCGACCACCAAGCGAAACATCGCATCGAGCGAGCACGTACTCGGATGGAA
GCCGGTCTTGTCGATCAGGATGATCTGGACGAAGAGCATCAGGGGCTCGCGCCAGCCGA
ACTGTTCGCCAGGCTCAAGGCGAGCATGCCCGACGGCGAGGATCTCGTCGTGACCCATG
GCGATGCCTGCTTGCCGAATATCATGGTGGAAAATGGCCGCTTTTCTGGATTCATCGACTG
TGGCCGGCTGGGTGTGGCGGACCGCTATCAGGACATAGCGTTGGCTACCCGTGATATTGC
TGAAGAGCTTGGCGGCGAATGGGCTGACCGCTTCCTCGTGCTTTACGGTATCGCCGCTCC
CGATTCGCAGCGCATCGCCTTCTATCGCCTTCTTGACGAGTTCTTCTGAGCGGGACTCTG
GGGTTCGAAATGACCGACCAAGCGACGCCCAACCTGCCATCACGAGATTTCGATTCCAC
CGCCGCCTTCTATGAAAGGTTGGGCTTCGGAATCGTTTTCCGGGACGCCGGCTGGATGAT
CCTCCAGCGCGGGGATCTCATGCTGGAGTTCTTCGCCCACCCTAGGGGGAGGCTAACTG
AAACACGGAAGGAGACAATACCGGAAGGAACCCGCGCTATGACGGCAATAAAAAGACA
GAATAAAACGCACGGTGTTGGGTCGTTTGTTCATAAACGCGGGGTTCGGTCCCAGGGCT
GGCACTCTGTCGATACCCCACCGAGACCCCATTGGGGCCAATACGCCCGCGTTTCTTCCT
TTTCCCCACCCCACCCCCCAAGTTCGGGTGAAGGCCCAGGGCTCGCAGCCAACGTCGGG
GCGGCAGGCCCTGCCATAGCCTCAGGTTACTCATATATACTTTAGATTGATTTAAAACTTC
ATTTTTAATTTAAAAGGATCTAGGTGAAGATCCTTTTTGATAATCTCATGACCAAAATCCCT
TAACGTGAGTTTTCGTTCCACTGAGCGTCAGACCCCGTAGAAAAGATCAAAGGATCTTCT
TGAGATCCTTTTTTTCTGCGCGTAATCTGCTGCTTGCAAACAAAAAAACCACCGCTACCA
GCGGTGGTTTGTTTGCCGGATCAAGAGCTACCAACTCTTTTTCCGAAGGTAACTGGCTTC
AGCAGAGCGCAGATACCAAATACTGTTCTTCTAGTGTAGCCGTAGTTAGGCCACCACTTC

AAGAACTCTGTAGCACCGCCTACATACCTCGCTCTGCTAATCCTGTTACCAGTGGCTGCT
GCCAGTGGCGATAAGTCGTGTCTTACCGGGTTGGACTCAAGACGATAGTTACCGGATAAG
GCGCAGCGGTCGGGCTGAACGGGGGGTTCGTGCACACAGCCCAGCTTGGAGCGAACGA
CCTACACCGAACTGAGATACCTACAGCGTGAGCTATGAGAAAGCGCCACGCTTCCCGAA
GGGAGAAAGGCGGACAGGTATCCGGTAAGCGGCAGGGTCGGAACAGGAGAGCGCACG
AGGGAGCTTCCAGGGGGAAACGCCTGGTATCTTTATAGTCCTGTCGGGTTTCGCCACCTC
TGACTTGAGCGTCGATTTTTGTGATGCTCGTCAGGGGGGCGGAGCCTATGGAAAAACGC
CAGCAACGCGGCCTTTTTACGGTTCCTGGCCTTTTGCTGGCCTTTTGCTCACATGTTCTTT
CCTGCGTTATCCCCTGATTCTGTGGATAACCGTATTACCGCCATGCAT

Donor Plasmid (PITCh hROSA26 pCAG-CYP2C9-P2A-CYP2D6-T2A-HygR-pA)

Full sequences of PITCh hROSA26 pCAG-CYP2C9-P2A-CYP2D6-T2A-HygR-pA donor plasmid is shown below.

PITCh gRNA target site, hROSA26 homology arm, CYP2C9, CYP2D6, HygR

gcatcgtacgcgtacgtgtttggaggggggaggggagtgccgcaatacctttatgggagttctctgctgcctcccgtcttgtaaggaccgccctgg
gcctggaagaagccctccctcctttcctTAGTTATTAATAGTAATCAATTACGGGGTCATTAGTTCATAGCC
CATATATGGAGTTCCGCGTTACATAACTTACGGTAAATGGCCCGCCTGGCTGACCGCCCAA
CGACCCCCGCCCATTGACGTCAATAATGACGTATGTTCCCATAGTAACGCCAATAGGGACT
TTCCATTGACGTCAATGGGTGGAGTATTTACGGTAAACTGCCCACTTGGCAGTACATCAA
GTGTATCATATGCCAAGTACGCCCCCTATTGACGTCAATGACGGTAAATGGCCCGCCTGGC
ATTATGCCCAGTACATGACCTTATGGGACTTTCCTACTTGGCAGTACATCTACGTATTAGTC
ATCGCTATTACCATGGTCGAGGTGAGCCCCACGTTCTGCTTCACTCTCCCCATCTCCCCCC
CCTCCCCACCCCCAATTTTGTATTTATTTATTTTTTAATTATTTTGTGCAGCGATGGGGGCG
GGGGGGGGGGGGGGCGCGCGCCAGGCGGGGCGGGGCGGGGCGAGGGGCGGGGCGG
GGCGAGGCGGAGAGGTGCGGCGGCAGCCAATCAGAGCGGCGCGCTCCGAAAGTTTCCT
TTTATGGCGAGGCGGCGGCGGCGGCGGCCCTATAAAAAGCGAAGCGCGCGGCGGGCGG
GAGTCGCTGCGCGCTGCCTTCGCCCCGTGCCCCGCTCCGCCGCCGCCTCGCGCCGCCCG
CCCCGGCTCTGACTGACCGCGTTACTCCCACAGGTGAGCGGGCGGGACGGCCCTTCTCC
TCCGGGCTGTAATTAGCGCTTGGTTTAATGACGGCTTGTTTCTTTTCTGTGGCTGCGTGAA
AGCCTTGAGGGGCTCCGGGAGGGCCCTTTGTGCGGGGGGAGCGGCTCGGGGGGTGCGT
GCGTGTGTGTGTGCGTGGGGAGCGCCGCGTGCGGCTCCGCGCTGCCCGGCGGCTGTGAG
CGCTGCGGGCGCGGCGCGGGGCTTTGTGCGCTCCGCAGTGTGCGCGAGGGGAGCGCGG
CCGGGGGCGGTGCCCCGCGGTGCGGGGGGGGCTGCGAGGGGAACAAAGGCTGCGTGC
GGGGTGTGTGCGTGGGGGGGTGAGCAGGGGGTGTGGGCGCGTCGGTCGGGCTGCAACC
CCCCCTGCACCCCCCTCCCCGAGTTGCTGAGCACGGCCCGGCTTCGGGTGCGGGGCTCC
GTACGGGGCGTGGCGCGGGGCTCGCCGTGCCGGGCGGGGGGTGGCGGCAGGTGGGGGT

GCCGGGCGGGGCGGGGCCGCCTCGGGCCGGGGAGGGCTCGGGGGAGGGGCGCGGCGG
CCCCCGGAGCGCCGGCGGCTGTCGAGGCGCGGCGAGCCGCAGCCATTGCCTTTTATGGT
AATCGTGCGAGAGGGCGCAGGGACTTCCTTTGTCCCAAATCTGTGCGGAGCCGAAATCT
GGGAGGCGCCGCCGCACCCCCTCTAGCGGGCGCGGGGCGAAGCGGTGCGGCGCCGGCA
GGAAGGAAATGGGCGGGGAGGGCCTTCGTGCGTCGCCGCGCCGCCGTCCCCTTCTCCCT
CTCCAGCCTCGGGGCTGTCCGCGGGGGGACGGCTGCCTTCGGGGGGGACGGGGCAGGG
CGGGGTTCGGCTTCTGGCGTGTGACCGGCGGCTCTAGAGCCTCTGCTAACCATGTTCATG
CCTTCTTCTTTTTCCTACAGCTCCTGGGCAACGTGCTGGTTATTGTGCTGTCTCATCATTTT
GGCAAAGAATTAACCCTCACTAAAGGGTACCGGGCCCCCCCTCGAGGTCGACGGTATC
GATAAGCTTGATATCGAATTgccgccatggattctcttgtggtccttgtgctctgtctctcatgtttgcttctcctttcactctggagac
agagctctgggagaggaaaactccctcctggccccactcctctcccagtgattggaaatatcctacagataggtattaaggacatcagcaaatcct
taaccaatctctcaaaggtctatggccctgtgttcactctgtattttggcctgaaacccatagtggtgctgcatggatatgaagcagtgaaggaagcc
ctgattgatcttggagaggagttttctggaagaggcattttcccactggctgaaagagctaacagaggatttggaattgttttcagcaatggaaagaa
atggaaggagatccggcgtttctccctcatgacgctgcggaattttgggatggggaagaggagcattgaggaccgtgttcaagaggaagcccgc
tgccttgtggaggagttgagaaaaaccaaggcctcaccctgtgatcccactttcatcctgggctgtgctccctgcaatgtgatctgctccattattttc
cataaacgttttgattataaagatcagcaatttcttaacttaatggaaaagttgaatgaaaacatcaagattttgagcagcccctggatccagatctgc
aataatttttctcctatcattgattacttcccgggaactcacaacaaattacttaaaaacgttgcttttatgaaaagttatattttggaaaaagtaaaagaa
caccaagaatcaatggacatgaacaaccctcaggactttattgattgcttcctgatgaaaatggagaaggaaaagcacaaccaaccatctgaattt
actattgaaagcttggaaaacactgcagttgacttgtttggagctgggacagagacgacaagcacaaccctgagatatgctctccttctcctgctga
agcacccagaggtcacagctaaagtccaggaagagattgaacgtgtgattggcagaaaccggagcccctgcatgcaagacaggagccacatg
ccctacacagatgctgtggtgcacgaggtccagagatacattgaccttctccccaccagcctgccccatgcagtgacctgtgacattaaattcaga
aactatctcattcccaagggcacaaccatattaatttccctgacttctgtgctacatgacaacaaagaatttcccaacccagagatgtttgaccctcat
cactttctggatgaaggtggcaattttaagaaaagtaaatacttcatgcctttctcagcaggaaaacggatttgtgtgggagaagccctggccggca
tggagctgtttttattcctgacctccattttacagaactttaacctgaaatctctggttgacccaaagaaccttgacaccactccagttgtcaatggattt
gcctctgtgccgcccttctaccagctgtgcttcattcctgtcggatctggagcaacaaacttctcactactcaaacaagcaggtgacgtggaggag
aatcccgggcctatggggctagaagcactggtgcccctggccgtgatagtggccatcttcctgctcctggtggacctgatgcaccggcgccaac
gctgggctgcacgctacccaccaggcccccctgccactgcccgggctgggcaacctgctgcatgtggacttccagaacacaccatactgcttcga
ccagttgcggcgccgcttcggggacgtgttcagcctgcagctggcctggacgccggtggtcgtgctcaatgggctggcggccgtgcgcgagg
cgctggtgacccacggcgaggacaccgccgaccgcccgcctgtgcccatcacccagatcctgggtttcgggccgcgttcccaaggggtgttcc
tggcgcgctatgggcccgcgtggcgcgagcagaggcgcttctccgtgtccaccttgcgcaacttgggcctgggcaagaagtcgctggagcagt
gggtgaccgaggaggccgcctgcctttgtgccgccttcgccaaccactccggacgcccctttcgccccaacggtctcttggacaaagccgtgag
caacgtgatcgcctccctcacctgcgggcgccgcttcgagtacgacgaccctcgcttcctcaggctgctggacctagctcaggagggactgaag
gaggagtcgggctttctgcgcgaggtgctgaatgctgtccccgtcctcctgcatatcccagcgctggctggcaaggtcctacgcttccaaaaggc
tttcctgacccagctggatgagctgctaactgagcacaggatgacctgggacccagcccagccccccgagacctgactgaggccttcctggca
gagatggagaaggccaaggggaaccctgagagcagcttcaatgatgagaacctgcgcatagtggtggctgacctgttctctgccgggatggtg
accacctcgaccacgctggcctggggcctcctgctcatgatcctacatccggatgtgcagcgccgtgtccaacaggagatcgacgacgtgatag

ggcaggtgcggcgaccagagatgggtgaccaggctcacatgccctacaccactgccgtgattcatgaggtgcagcgctttggggacatcgtcc
ccctgggtgtgacccatatgacatcccgtgacatcgaagtacagggcttccgcatccctaagggaacgacactcatcaccaacctgtcatcggtg
ctgaaggatgaggccgtctgggagaagcccttccgcttccaccccgaacacttcctggatgcccagggccactttgtgaagccggaggccttcc
tgcctttctcagcaggccgccgtgcatgcctcggggagcccctggcccgcatggagctcttcctcttcttcacctccctgctgcagcacttcagctt
ctcggtgcccactggacagccccggcccagccaccatggtgtctttgctttcctggtgagcccatccccctatgagctttgtgctgtgccccgcgg
cagtggagagggcagaggaagtctgctaacatgcggtgacgtcgaggagaatcctggcccaatgaaaaagcctgaactcaccgcgacgtctgt
cgagaagtttctgatcgaaaagttcgacagcgtttccgacctgatgcagctctcggagggcgaagaatctcgtgctttcagcttcgatgtaggagg
gcgtggatatgtcctgcgggtaaatagctgcgccgatggtttctacaaagatcgttatgtttatcggcactttgcatcggccgcgctcccgattccgg
aagtgcttgacattggggaattcagcgagagcctgacctattgcatctcccgccgtgcacagggtgtcacgttgcaagacctgcctgaaaccgaa
ctgcccgctgttctgcagccggtcgcggaggccatggatgcgatcgctgcggccgatcttagccagacgagcgggttcggcccattcggaccg
caaggaatcggtcaatacactacatggcgtgatttcatatgcgcgattgctgatccccatgtgtatcactggcaaactgtgatggacgacaccgtca
gtgcgtccgtcgcgcaggctctcgatgagctgatgctttgggccgaggactgccccgaagtccggcacctcgtgcacgcggatttcggctccaa
caatgtcctgacggacaatggccgcataacagcggtcattgactggagcgaggcgatgttcggggattcccaatacgaggtcgccaacatcttct
tctggaggccgtggttggcttgtatggagcagcagacgcgctacttcgagcggaggcatccggagcttgcaggatcgccgcggctccgggcgt
atatgctccgcattggtcttgaccaactctatcagagcttggttgacggcaatttcgatgatgcagcttgggcgcagggtcgatgcgacgcaatcgt
ccgatccggagccgggactgtcgggcgtacacaaatcgcccgcagaagcgcggccgtctggaccgatggctgtgtagaagtactcgccgata
gtggaaaccgacgccccagcactcgtccgagggcaaaggaatagGGATCCACTAGTTCTAGAGCGGCCGCGACT
CTAGATCATAATCAGCCATACCACATTTGTAGAGGTTTTACTTGCTTTAAAAAACCTCCCA
CACCTCCCCCTGAACCTGAAACATAAAATGAATGCAATTGTTGTTGTTAACTTGTTTATTG
CAGCTTATAATGGTTACAAATAAAGCAATAGCATCACAAATTTCACAAATAAAGCATTTTT
TTCACTGCATTCTAGTTGTGGTTTGTCCAAACTCATCAATGTATCTTActcgcgtgatctcgtcatcgcc
tccatgtcgagtcgcttctcgattatgggcgggattcttttgcctaggcttaaggggctaacttggtccctgggcgttccaaacacgtacgcgtacga
tgcAGGCGTAAATTGTAAGCGTTAATATTTTGTTAAAATTCGCGTTAAATTTTTGTTAAATCA
GCTCATTTTTTAACCAATAGGCCGAAATCGGCAAAATCCCTTATAAATCAAAAGAATAGAC
CGAGATAGGGTTGAGTGTTGTTCCAGTTTGGAACAAGAGTCCACTATTAAAGAACGTGG
ACTCCAACGTCAAAGGGCGAAAAACCGTCTATCAGGGCGATGGCCCACTACGTGAACCA
TCACCCTAATCAAGTTTTTTGGGGTCGAGGTGCCGTAAAGCACTAAATCGGAACCCTAAA
GGGAGCCCCCGATTTAGAGCTTGACGGGGAAAGCCGGCGAACGTGGCGAGAAAGGAAG
GGAAGAAAGCGAAAGGAGCGGGCGCTAGGGCGCTGGCAAGTGTAGCGGTCACGCTGCG
CGTAACCACCACACCCGCCGCGCTTAATGCGCCGCTACAGGGCGCGTCAGGTGGCACTT
TTCGGGGAAATGTGCGCGGAACCCCTATTTGTTTATTTTTCTAAATACATTCAAATATGTAT
CCGCTCATGAGACAATAACCCTGATAAATGCTTCAATAATATTGAAAAAGGAAGAGTCCT
GAGGCGGAAAGAACCAGCTGTGGAATGTGTGTCAGTTAGGGTGTGGAAAGTCCCCAGG
CTCCCCAGCAGGCAGAAGTATGCAAAGCATGCATCTCAATTAGTCAGCAACCAGGTGTG
GAAAGTCCCCAGGCTCCCCAGCAGGCAGAAGTATGCAAAGCATGCATCTCAATTAGTCA
GCAACCATAGTCCCGCCCCTAACTCCGCCCATCCCGCCCCTAACTCCGCCCAGTTCCGCC

CATTCTCCGCCCCATGGCTGACTAATTTTTTTTATTTATGCAGAGGCCGAGGCCGCCTCGG
CCTCTGAGCTATTCCAGAAGTAGTGAGGAGGCTTTTTTGGAGGCCTAGGCTTTTGCAAAG
ATCATCAAGAGACAGGATGAGGATCGTTTCGCATGATTGAACAAGATGGATTGCACGCAG
GTTCTCCGGCCGCTTGGGTGGAGAGGCTATTCGGCTATGACTGGGCACAACAGACAATC
GGCTGCTCTGATGCCGCCGTGTTCCGGCTGTCAGCGCAGGGGCGCCCGGTTCTTTTTGTC
AAGACCGACCTGTCCGGTGCCCTGAATGAACTGCAAGACGAGGCAGCGCGGCTATCGTG
GCTGGCCACGACGGGCGTTCCTTGCGCAGCTGTGCTCGACGTTGTCACTGAAGCGGGAA
GGGACTGGCTGCTATTGGGCGAAGTGCCGGGGCAGGATCTCCTGTCATCTCACCTTGCTC
CTGCCGAGAAAGTATCCATCATGGCTGATGCAATGCGGCGGCTGCATACGCTTGATCCGG
CTACCTGCCCATTCGACCACCAAGCGAAACATCGCATCGAGCGAGCACGTACTCGGATG
GAAGCCGGTCTTGTCGATCAGGATGATCTGGACGAAGAGCATCAGGGGCTCGCGCCAGC
CGAACTGTTCGCCAGGCTCAAGGCGAGCATGCCCGACGGCGAGGATCTCGTCGTGACCC
ATGGCGATGCCTGCTTGCCGAATATCATGGTGGAAAATGGCCGCTTTTCTGGATTCATCGA
CTGTGGCCGGCTGGGTGTGGCGGACCGCTATCAGGACATAGCGTTGGCTACCCGTGATAT
TGCTGAAGAGCTTGGCGGCGAATGGGCTGACCGCTTCCTCGTGCTTTACGGTATCGCCGC
TCCCGATTCGCAGCGCATCGCCTTCTATCGCCTTCTTGACGAGTTCTTCTGAGCGGGACTC
TGGGGTTCGAAATGACCGACCAAGCGACGCCCAACCTGCCATCACGAGATTTCGATTCC
ACCGCCGCCTTCTATGAAAGGTTGGGCTTCGGAATCGTTTTCCGGGACGCCGGCTGGATG
ATCCTCCAGCGCGGGGATCTCATGCTGGAGTTCTTCGCCCACCCTAGGGGGAGGCTAACT
GAAACACGGAAGGAGACAATACCGGAAGGAACCCGCGCTATGACGGCAATAAAAAGAC
AGAATAAAACGCACGGTGTTGGGTCGTTTGTTCATAAACGCGGGGTTCGGTCCCAGGGC
TGGCACTCTGTCGATACCCCACCGAGACCCCATTGGGGCCAATACGCCCGCGTTTCTTCC
TTTTCCCCACCCCACCCCCCAAGTTCGGGTGAAGGCCCAGGGCTCGCAGCCAACGTCGG
GGCGGCAGGCCCTGCCATAGCCTCAGGTTACTCATATATACTTTAGATTGATTTAAAACTT
CATTTTTAATTTAAAAGGATCTAGGTGAAGATCCTTTTTGATAATCTCATGACCAAAATCC
CTTAACGTGAGTTTTCGTTCCACTGAGCGTCAGACCCCGTAGAAAAGATCAAAGGATCTT
CTTGAGATCCTTTTTTTCTGCGCGTAATCTGCTGCTTGCAAACAAAAAAACCACCGCTAC
CAGCGGTGGTTTGTTTGCCGGATCAAGAGCTACCAACTCTTTTTCCGAAGGTAACTGGCT
TCAGCAGAGCGCAGATACCAAATACTGTTCTTCTAGTGTAGCCGTAGTTAGGCCACCACT
TCAAGAACTCTGTAGCACCGCCTACATACCTCGCTCTGCTAATCCTGTTACCAGTGGCTGC
TGCCAGTGGCGATAAGTCGTGTCTTACCGGGTTGGACTCAAGACGATAGTTACCGGATAA
GGCGCAGCGGTCGGGCTGAACGGGGGGTTCGTGCACACAGCCCAGCTTGGAGCGAACG
ACCTACACCGAACTGAGATACCTACAGCGTGAGCTATGAGAAAGCGCCACGCTTCCCGA
AGGGAGAAAGGCGGACAGGTATCCGGTAAGCGGCAGGGTCGGAACAGGAGAGCGCAC
GAGGGAGCTTCCAGGGGGAAACGCCTGGTATCTTTATAGTCCTGTCGGGTTTCGCCACCT
CTGACTTGAGCGTCGATTTTTGTGATGCTCGTCAGGGGGGCGGAGCCTATGGAAAAACG

CCAGCAACGCGGCCTTTTTACGGTTCCTGGCCTTTTGCTGGCCTTTTGCTCACATGTTCTT
TCCTGCGTTATCCCCTGATTCTGTGGATAACCGTATTACCGCCATGCAT
